# A multipeptide vaccine plus toll-like receptor agonists LPS or polyICLC in combination with incomplete Freund’s adjuvant in melanoma patients

**DOI:** 10.1186/s40425-019-0625-x

**Published:** 2019-06-27

**Authors:** Marit M. Melssen, Gina R. Petroni, Kimberly A. Chianese-Bullock, Nolan A. Wages, William W. Grosh, Nikole Varhegyi, Mark E. Smolkin, Kelly T. Smith, Nadejda V. Galeassi, Donna H. Deacon, Elizabeth M. Gaughan, Craig L. Slingluff

**Affiliations:** 1 Department of Surgery/Division of Surgical Oncology and the Human Immune Therapy Center, Cancer Center, University of Virginia, 1352 Pinn Hall, P.O. Box 801457, Charlottesville, VA 22908 USA; 2 Department of Public Health Sciences/Division of Translational Research & Applied Statistics, University of Virginia, Charlottesville, VA USA; 3 Department of Medicine/Division of Hematology/Oncology, University of Virginia, Charlottesville, VA USA; 4 Department of Microbiology, Immunology, and Cancer Biology, University of Virginia, Charlottesville, VA USA

**Keywords:** Melanoma, Peptide vaccine, Incomplete freund’s adjuvant, Toll-like receptor, Lipopolysaccharide, polyICLC, Clinical trial, CD8 T cells, Immune response, ELIspot

## Abstract

**Background:**

Cancer vaccines require adjuvants to induce effective immune responses; however, there is no consensus on optimal adjuvants. We hypothesized that toll-like receptor (TLR)3 agonist polyICLC or TLR4 agonist lipopolysaccharide (LPS), combined with CD4 T cell activation, would support strong and durable CD8^+^ T cell responses, whereas addition of an incomplete Freund’s adjuvant (IFA) would reduce magnitude and persistence of immune responses.

**Patients and methods:**

Participants with resected stage IIB-IV melanoma received a vaccine comprised of 12 melanoma peptides restricted by Class I MHC (12MP), plus a tetanus helper peptide (Tet). Participants were randomly assigned 2:1 to cohort 1 (LPS dose-escalation) or cohort 2 (polyICLC). Each cohort included 3 subgroups (a-c), receiving 12MP + Tet + TLR agonist without IFA (0), or with IFA in vaccine one (V1), or all six vaccines (V6). Toxicities were recorded (CTCAE v4). T cell responses were measured with IFNγ ELIspot assay ex vivo or after one in vitro stimulation (IVS).

**Results:**

Fifty-three eligible patients were enrolled, of which fifty-one were treated. Treatment-related dose-limiting toxicities (DLTs) were observed in 0/33 patients in cohort 1 and in 2/18 patients in cohort 2 (11%). CD8 T cell responses to 12MP were detected ex vivo in cohort 1 (42%) and in cohort 2 (56%) and in 18, 50, and 72% for subgroups V0, V1, and V6, respectively. T cell responses to melanoma peptides were more durable and of highest magnitude for IFA V6.

**Conclusions:**

LPS and polyICLC are safe and effective vaccine adjuvants when combined with IFA. Contrary to the central hypothesis, IFA enhanced T cell responses to peptide vaccines when added to TLR agonists. Future studies will aim to understand mechanisms underlying the favorable effects with IFA.

**Trial registration:**

The clinical trial Mel58 was performed with IRB (#15781) and FDA approval and is registered with Clinicaltrials.gov on April 25, 2012 (NCT01585350). Patients provided written informed consent to participate. Enrollment started on June 24, 2012.

**Electronic supplementary material:**

The online version of this article (10.1186/s40425-019-0625-x) contains supplementary material, which is available to authorized users.

## Introduction

Resistance to checkpoint blockade immunotherapy is commonly attributed to a lack of pre-existing T cell responses to cancer antigens. Thus, there is compelling need for methods to induce antitumor immunity. Cancer vaccines targeting either mutated neo-antigens or shared tumor antigens may accomplish this; however, a critical limitation of cancer vaccine technology is lack of consensus on optimal vaccine adjuvants, which are required to induce functional immune responses. Clinical trials to test adjuvants are more feasible with shared antigen vaccines than with mutated neo-antigens because neo-antigen vaccine composition varies for each patient, whereas the composition of a shared antigen vaccine is consistent across the study population.

The most common adjuvant for peptide vaccines in melanoma has been an incomplete Freund’s adjuvant (IFA). Peptide vaccines incorporating IFA have induced circulating T cell responses [1–3], but some are weak and transient [4]. Recent studies in mice have shown negative effects of IFA as a vaccine adjuvant [5, 6] and have suggested instead that an optimal adjuvant for short peptide vaccines is a TLR agonist plus an agonistic CD40 antibody, which induced strong and durable T cell responses and tumor control [5]. A goal of the present trial was to evaluate a similar approach in patients with melanoma. A multipeptide vaccine (12MP) has previously been found to be both safe and immunogenic [7, 8]. When this trial was initiated, agonistic CD40 antibodies were not available for clinical use. Instead, we used an alternative approach to support licensing of antigen presenting cells (APC) through CD40. Activated CD4 T cells upregulate CD40L; so, we included a peptide from tetanus toxoid known to activate CD4 T cells at the vaccine site and draining node [9–11]. We have shown that a modified form of the p2 peptide of tetanus toxoid residues 830–844 (AQYIKANSKFIGITEL, Tet) induces strong CD4 T cell responses in patients [8, 12]; so, inclusion of this peptide may offer an alternative to CD40 antibodies. Thus, the present study was designed to evaluate the safety and immunogenicity of vaccinating with a mixture of 12 short melanoma peptides (12MP) plus a tetanus helper peptide, combined with TLR agonists. To assess whether IFA interferes with vaccine activity, the study also included treatment arms with IFA.

The central hypotheses were that the TLR agonists may be safe and effective vaccine adjuvants and that decreasing use of IFA may further enhance the magnitude and persistence of the immune responses. Specific goals were: a) to determine the safety of intradermal and subcutaneous injection of the TLR4 agonist lipopolysaccharide (LPS) as a vaccine adjuvant with a multipeptide vaccine, b) to obtain preliminary data on whether administration of a multipeptide vaccine plus each of 2 TLR agonists is immunogenic with or without IFA, c) to obtain preliminary data on whether addition of either of two TLR agonists improves the persistence of circulating CD8 T cell responses to vaccination with a multipeptide vaccine, and d) to determine the local and systemic toxicities of administration of a multipeptide vaccine with each of 2 TLR agonists, and with or without IFA.

## Materials and methods

### Patient eligibility

Patients at least 18 years of age, expressing HLA-A1, −A2, −A3, −A11 or -A31 were eligible if they had biopsy-proven Stage IIB-IV melanoma rendered clinically free of disease by surgery, other therapy or spontaneous remission. Patients with Stage III-IV melanoma with definite or equivocal findings of persistent metastatic disease could be eligible if they did not meet RECIST criteria for measurable disease. Also required were ECOG performance status (PS) 0–1, and adequate organ function.

### Vaccine components and treatment regimen

**Fig. 1:**
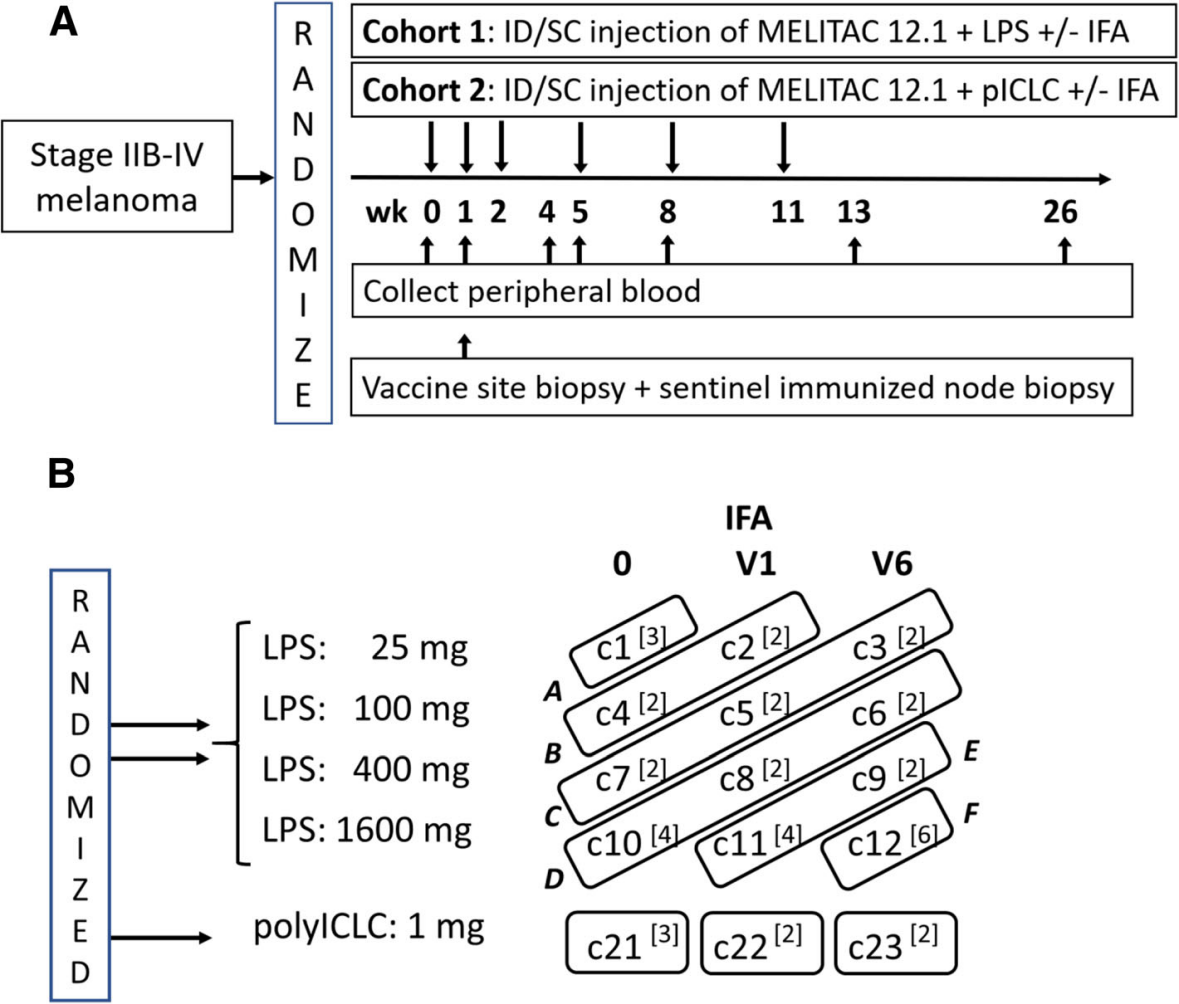
Clinical trial design. The schema for the clinical trial is shown in **a**. The zones for dose escalation of LPS in cohort 1 (A-F) are shown in **b**. The study combinations are numbered c1 - c12 for cohort 1 and c21-c23 for cohort 2 as shown

All participants were vaccinated with MELITAC 12.1 (100mcg of each of 12 Class I MHC restricted melanoma peptides (12MP) [7] and 200mcg of a tetanus helper peptide [12] (Additional file 1: Table S1)). Vaccines were administered with either of two TLR agonists and with or without IFA (Fig. 1a). The IFA used was Montanide ISA-51VG adjuvant (Seppic, Inc., Puteaux, France). PolyICLC (lot PJ2515-1-10, 2.0 mg/ml dry weight) was provided by the Cancer Research Institute/Ludwig Institute for Cancer Research (New York), who purchased it from Oncovir (Washington, DC). LPS was provided by Dr. Anthony Suffredini (Drug Master File Number BB-MF7294) at the National Cancer Institute (NCI) and was vialed and tested by Cambrex BioScience (Walkersville, MD) under oversight of the Biopharmaceutical Development Program, SAIC-Frederick, Inc., NCI-Frederick, Frederick, MD. Each vial contained lyophilized solid representing 10,000 endotoxin units (EU) of *E. coli* O:113 Reference Endotoxin Lot CC-RE-LOT 3 (1mcg endotoxin). Upon reconstitution in 5 mL water, it contained 2000 EU/mL in 1% Lactose, 0.1% PEG-6000. Regimens were administered half-subcutaneously and half-intradermally in one skin location that is rotated to different extremity sites on days 1, 8, 15, 36, 57 and 78.

### Study design

This was an early phase trial designed to determine the maximum tolerated dose combination (MTDC) of LPS and IFA from among twelve possible combinations in cohort 1 and the MTDC of polyICLC and IFA from among three possible combinations in cohort 2, and to obtain preliminary data on immune response for all the combinations under study. Eligible patients were randomly assigned 2:1 to cohort 1 or cohort 2 (Fig. 1). The 12 combinations in cohort 1 included 4 dose levels of endotoxin (25, 100, 400, 1600EU) administered in three vaccine regimens (12MP + Tet + LPS) and i) without IFA (V0), ii) plus IFA in the first vaccine only (V1), or iii) plus IFA in all six vaccines (V6); (Fig. 1b, Additional file 1: Table S2). The 3 combinations in cohort 2 included 1 dose level of polyICLC (1 mg) administered similarly for each of the three adjuvant regimens and, V0 or V1 or V6; (Fig. 1b, Additional file 1: Table S2). Toxicities were recorded (CTCAE v4). Blood was collected weeks 0, 1, 4, 5, 8, 13, and 26 (Fig. 1a). One week after the first vaccine, a vaccine site-draining lymph node was harvested under local anesthesia, using techniques reported [13], and 4 mm punch biopsies of that vaccine site were obtained (Fig. 1a).

In cohort 1, dose escalation was conducted using a two-stage method for dose-finding for combinations of agents [14]. The first stage was designed such that participants were treated in groups of size 2 until a participant experienced a dose-limiting toxicity (DLT), after which a model-based allocation (stage 2) began. The escalation plan for the first stage was based on grouping dose combinations into “zones,” which are shown in Fig. 1b and detailed in Additional file 2: Supplemental Text. With this dose escalation design, participants were accrued and assigned to other open combinations within a zone, but escalation did not occur outside the zone until a minimum 3-week follow-up period was observed for the first 2 participants accrued to a combination. The second stage modeling strategy using the continual reassessment model (CRM) [15] was planned but not realized since no participants in cohort 1 experienced a DLT. Additional design details are provided in Additional file 1: Supplemental Text. For cohort 2, with only 3 possible combinations of interest, the goal was to accrue 3 patients per combination in increasing magnitude conditional on 1 or fewer DLTs being observed and then to randomly accrue up to 3 additional patients per combination (Fig. 1b).

A DLT was defined as any unexpected adverse event that was possibly, probably, or definitely related to treatment and (1) ≥ Grade 1 selected ocular adverse events, (2) ≥ Grade 2 allergic reactions, (3) ≥ Grade 3 non-hematologic/non-metabolic toxicities, and (4) ≥ Grade 3 hematologic/metabolic toxicities. Grade 2 nausea and Grade 3 fatigue lasting ≤3 days after vaccination were expected toxicities, and injection site ulceration was expected in a subset of patients but vaccine site ulceration of 2 cm diameter or greater was considered a DLT.

### Expansion

To assess the impact of including IFA (or not) on the immunologic parameters, the goal was to accrue up to 6 patients at the highest levels of LPS considered safe for each level of IFA. The choice of 6 patients per final combination was chosen to provide improved estimates of variability.

### ELIspot assays

T cell responses were measured with IFNγ ELIspot either directly ex vivo, after cryopreservation (direct) or after in vitro sensitization (IVS). Responses to the 12 class I MHC-restricted melanoma peptides are mediated by CD8 T cells specifically [16–26], and responses to the tetanus peptide are mediated by CD4 T cells [12, 27]. Therefore, total PBMC were used for ELIspot assays, and responses per CD8 and CD4 counts were calculated based on their proportion of total PBMC as determined by flow cytometry as previously reported [8, 28, 29]. Methods for the IVS ELIspot assay have been reported [28]. For direct ELIspot assays, 200,000 PBMC were plated per well, and pulsed with synthetic peptide (10mcg/ml), in quadruplicate. Controls included irrelevant peptides, a mixture of viral peptides (CEF peptide pool), PMA-ionomycin and PHA. Evaluation of T-cell responses was based on the following definitions:

N_vax_ = number T-cells responding to vaccine peptide; N_neg_ = number T-cells responding to maximum negative control; R_vax_ = N_vax_/N_neg_. For evaluations of PBMC, a patient was considered to have a T-cell response to vaccination (binary yes/no), by direct ELIspot assay only if all the following criteria were met: (1) N_vax_ exceeded N_neg_ by at least 20/100,000 CD4 or CD8 cells (0.02%), where CD8 and CD4 counts were based on flow cytometry of PBMC. (2) R_vax_ ≥ 2, (3) (N_vax_–1SD) ≥ (N_neg_ + 1SD), and (4) R_vax_ after vaccination ≥2 x R_vax_ pre-vaccine, as described in our prior analyses [8, 28]. The same criteria applied for IVS ELIspot assays except that the threshold for criterion (1) was higher at 30/100,000 CD8 cells. Fold-increases less than one were set to one to indicate no response and to prevent overinflating adjusted fold-increases. Continuous measures of immune response denoted as fold-increase must satisfy conditions (1)–(3) and were defined as the amount of R_vax_.

Interassay coefficients of variation (CVs) were calculated for the response of 2 normal donors to the CEF peptide pool: for the high responder, mean number of spots per 100,000 cells was 250, and CV was 30%, and for the low responder, mean was 40 and CV was 44%.

### Statistical analysis of immunologic analyses

Primary immunologic analyses were based upon eligible patients, and maximal immune response was based upon responses in the blood through week 26. For hypothesis-testing, patients who discontinued protocol therapy prior to collection of all blood samples for allergic reactions or adverse events, disease progression, or noncompliance were considered immune response failures if no response was observed in evaluable samples. Immune response was a binary indicator of whether or not the criteria listed above were met, and immune response rates were calculated as the proportion of participants with an immune response. Point estimates and 90% confidence intervals were calculated for all summary parameters. Permutation tests [30] were used to assess differences in number of T-cells responding to vaccine peptide adjusting for negative control (i.e., N_vax_-N_neg_) over the first 12 weeks across groups defined by combinations of LPS dose, inclusion of IFA and inclusion of polyICLC. *P*-values were based upon 2000 randomly generated permutations and a *p*-value cutoff of 10% was used to indicate statistically significant results. Negative binomial regression was used to assess count data and contrasts were used to test specific hypotheses with *p*-values computed from the likelihood ratio chi-square test statistic (LR).

## Results

### Clinical characteristics

Total enrollment was 53 participants; however, 2 participants did not receive study treatment. Thus, demographic, safety, and immunologic summary data are reported for 51 patients who were enrolled and treated. These included 33 males (65%) and 18 females (35%). Most patients had ECOG PS of 0 (90%) and stage III disease at registration (78%). Additional details are provided in Additional file 1: Table S3.

### Toxicities and adverse events

Treatment related adverse events (AE) were limited to grades 1–3, with only one grade 3 (Additional file 1: Table S4). Two participants experienced DLTs, both in cohort 2 (polyICLC). One treated on the V1 sub-arm had grade 3 skin ulceration and was taken off study after 3 vaccines. One on the V6 sub-arm experienced several grade 2 toxicities, none of which individually met predefined criteria for a DLT, but which in aggregate were felt to be dose-limiting. This patient was taken off treatment after 4 vaccines. Overall, no study combinations were estimated to be too toxic for patient accrual.

### CD8 T cell response to 12MP

**Fig. 2:**
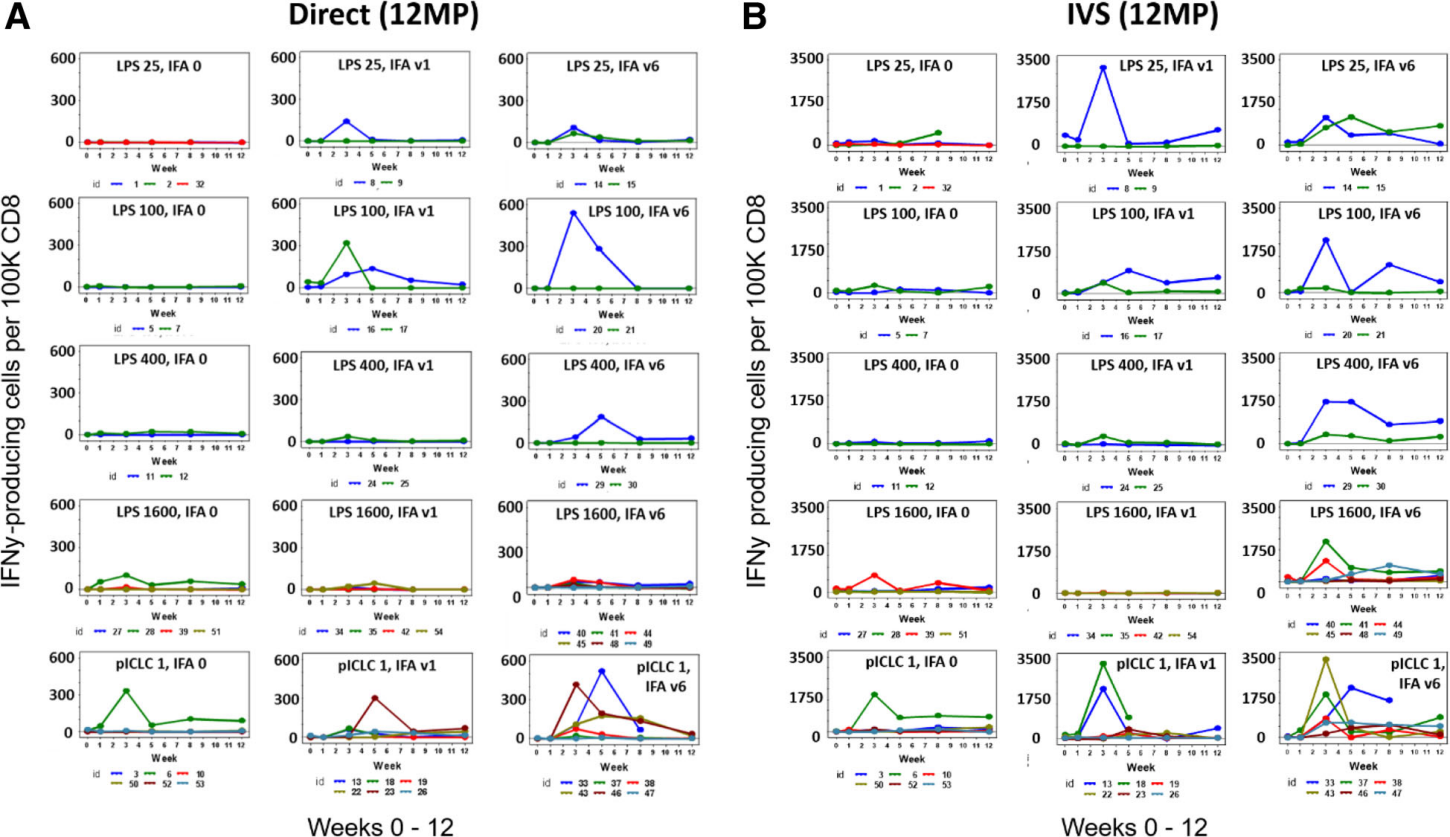
T cell responses over time (weeks 0–12). CD8 T cell responses to 12MP are shown for each patient from direct ELIspot assays (**a**), and from IVS ELIspot assays (**b**). Direct assay data represent response to pooled 12MP; IVS ELIspot data represent sum of responses to each of the 12 individual peptides. Response magnitude is shown as the number of IFNγ-secreting cells, less negative controls, per 100,000 CD8 cells. Values are shown as zero if they did not meet criteria for positivity

T cell responses to 12 peptide epitopes were evaluated both against the pool of 12 peptides (12MP pool), as well as each peptide individually, using IFNγ ELIspot assays. As described in the methods section, pre-existing immune responses were not considered responses to vaccination: in the uncommon cases with pre-existing immune responses, response to vaccination required at least a 2-fold increase over pre-existing response. The primary comparisons among study groups were made for weeks 0–12, as these data were consistently available (Fig. 2a). Responses to 12MP were detected ex vivo for 47% of patients overall, with the magnitude exceeding 600 spots/10^5^ CD8 T cells for some patients (Fig. 2a). Responses per study cohort and sub-arm are summarized in Additional file 1: Table S5 and per patient in Additional file 1: Table S6. Ex vivo T cell responses to 12MP were detected in 14 of 33 patients (42%) in cohort 1 (LPS) and in 10 of 18 patients (56%) for cohort 2 (polyICLC). Overall, for study arms with no IFA; IFA V1, and IFA V6, CD8 T cell responses to 12MP were detected ex vivo in 18, 50, and 72% of patients, respectively. Similarly, the sum of CD8 T cell responses to each of the 12 peptides was assessed after IVS, and these plots are shown in Fig. 2b.

Patterns of immune response over time were compared across study groups by modeling the data in PBMC across all time points through week 12. This method is a statistically robust assessment of differences in response patterns between groups over time (Table 1). In cohort 2 (polyICLC), direct ELIspot responses to 12MP were higher if IFA was given for all vaccines compared to no IFA (V6 vs V0, *p* = 0.036). This was evident also for cohort 1 (*p* = 0.065) and for analysis across both cohorts (p = 0.036). The CD8 response to 12MP also was higher with polyICLC than with the highest dose of LPS, among patients receiving IFA with all 6 vaccines (*p* = 0.031). Similarly, the ex vivo and IVS CD8 responses to the sum of individual peptides in 12MP were higher for polyICLC than for LPS1600 and for V6 than V0 in multiple comparisons (Table 1). Thus, for both direct and IVS ELIspot assessments, polyICLC was a more effective adjuvant than LPS, and inclusion of IFA in all vaccines significantly enhanced CD8 T cell response rates to defined melanoma antigens.

**Table 1:**
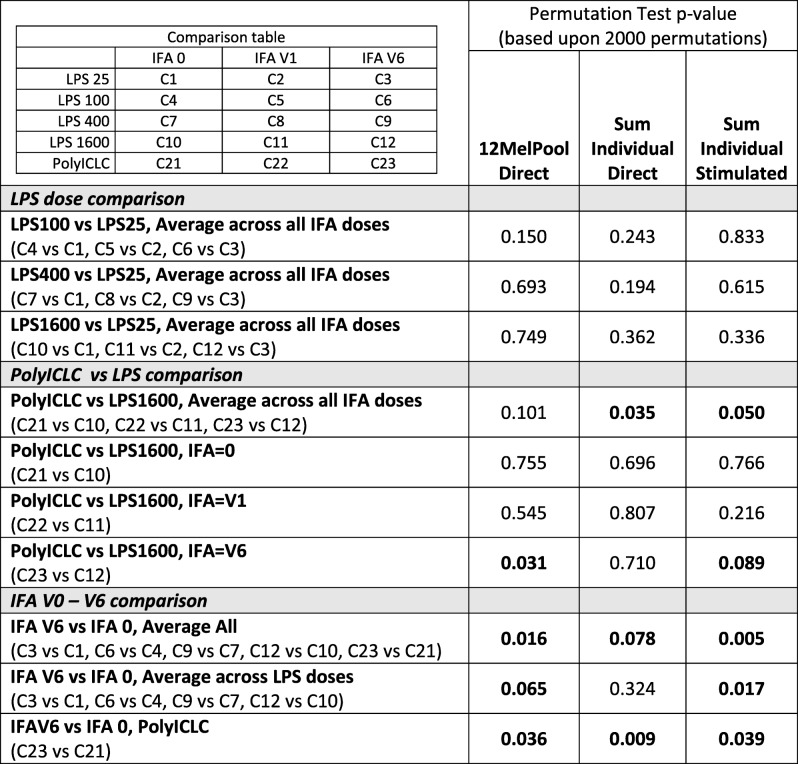
MEL58 ELIspot data comparisons across time (weeks 0–12)

Bolded numbers represent *P*-values of less than 0.1

We also evaluated immune responses in the sentinel immunized nodes (SINs), but the SINs were harvested early (week 1), and responses were not detected ex vivo. However, among 34 patients evaluated for immune response in the SIN after in vitro stimulation, 11 (32%) had an immune response. These included 18% (4/18) after vaccines with LPS, and 58% (7/12) after vaccines with pICLC. Immune responses in the SIN were observed in 27% (3/11) without IFA, and in 35% (8/23) with IFA (V1 or V6). These SIN responses are shown for all patients in Additional file 1: Table S6.

A pre-existing T cell response to 12MP was detected ex vivo in only 1 patient (#53), who did not develop a vaccine-induced T cell response. In IVS ELIspot assays, 3 (6%) had small pre-existing responses (patients 1, 14, 28), of whom 2 developed vaccine induced responses to 12MP ex vivo, and 2 had responses to 12MP after in vitro stimulation. As specified in the methods, a vaccine-induced T cell response was reported only if there was additional response of at least 2x any pre-existing response.

For the two patients in cohort 2 who came off early for DLTs, immune response data are shown in Additional file 1: Figure S1, where T cell responses to multiple peptides were evident in both.

### CD4^+^ T cell responses to tetanus peptide

T cell responses to the tetanus helper peptide were assessed in direct ELIspot assays. Overall, the permutation tests found no significant differences in response patterns to tetanus peptide among cohorts or study arms. Individual plots of these data for all patients are shown in Additional file 1: Figure S2. T cell responses to the tetanus peptide for any time point were observed in 58% (90% CI:[42, 72]) of patients on cohort 1 and 72% (90% CI:[50, 88]) on cohort 2, and in 24% (90% CI:[8, 46]), 75% (90% CI:[52, 91]), and 89% (90% CI:[69, 98]) of patients in subgroups V0, V1, and V6, respectively (Additional file 1: Table S5).

### Magnitude and breadth of CD8 T cell responses

**Fig. 3:**
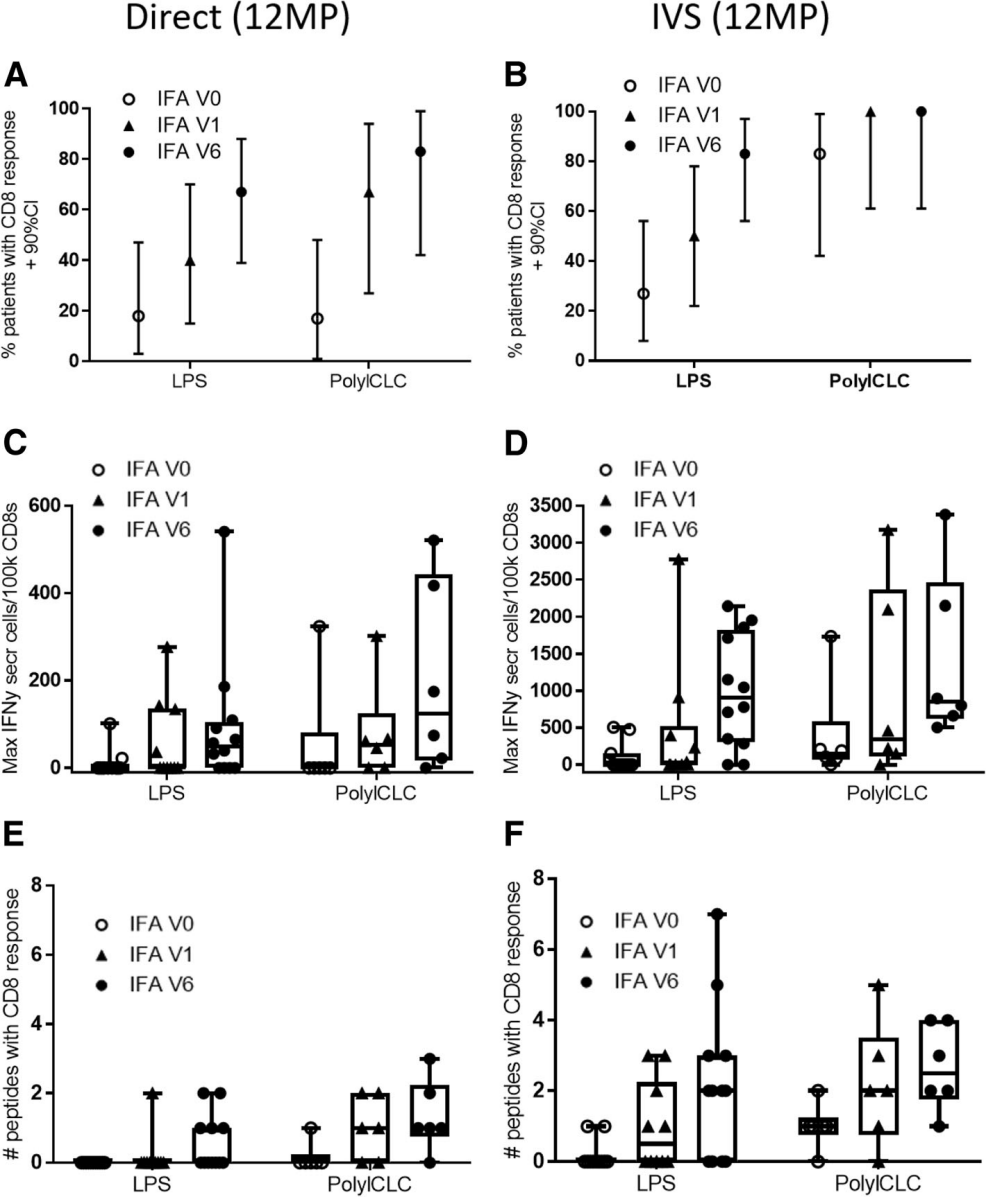
Frequency and Magnitude of T cell responses to 12MP by ELIspot assay ex vivo (**a, c, e**), and after IVS (**b, d, f**). The proportion and 90% confidence interval (CI) of patients with a response to 12MP pool are shown in panels A and B, for each cohort and subgroup. The magnitude of these responses (maximum number of spots per 1 × 10^5^ CD8 T cells) is shown in (**c**) and (**d**), where each symbol represents the maximum response for a patient. If the values did not meet criteria for a response, they are shown as zero**.** Boxplots represent 25th to 75th percentiles, with tails showing the full range, except outliers. The number of peptides to which a response was detected is shown for each patient with a response ex vivo (**e**) and after IVS (**f**)

In addition to modeling the immune response across the study population, the fractions of patients with CD8 T cell responses were assessed directly. Immune response rates to 12MP increased with increasing IFA use, both in direct and IVS ELIspot assays (Fig. 3a, b). Similar findings were evident with direct ELIspot based on the sum of responses to the individual peptides (Additional file 1: Table S5), with higher responses for cohort 2 than cohort 1. When IFA was included, the maximum number of IFNγ-secreting cells was higher in direct (Fig. 3c) and in IVS ELIspot assays (Fig. 3d). Similarly, the fold-increase in the T cell responses to 12MP was also higher with inclusion of IFA (data not shown). Immune responses were detected to a broader range of peptides when IFA was included in ex vivo assays (Fig. 3e) or in IVS assays (Fig. 3f).

### Persistence and durability of the CD8 immune responses

**Fig. 4:**
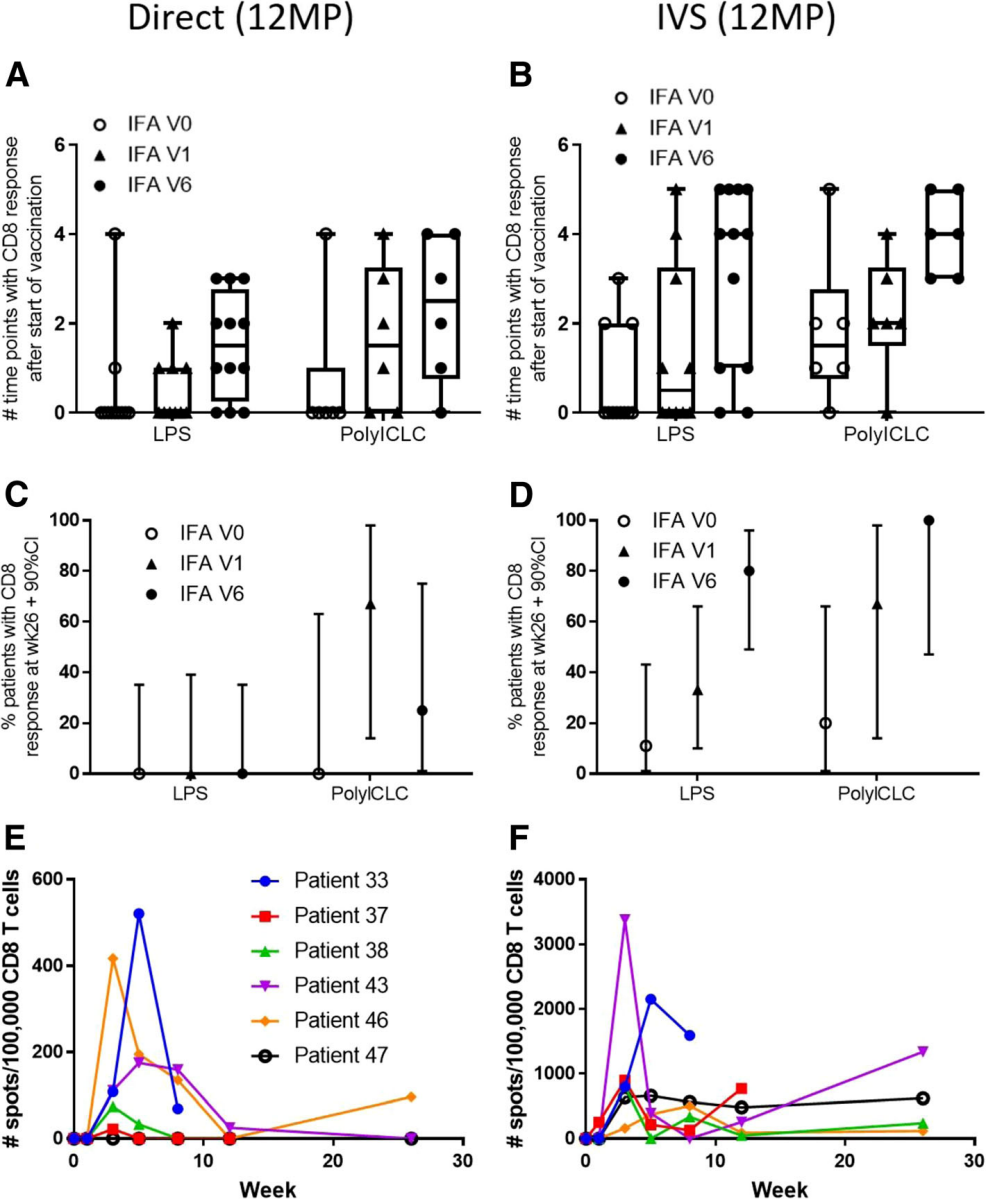
Persistence and durability of the CD8 T cell responses to 12MP. Persistence of the T cell responses to 12MP are shown in **a** (ex vivo) and **b** (IVS) as the number of PBMC dates in which a response was detected (after week 0). The maximum possible is 6 (after baseline). Durability of the T cell response for 3 months after the last vaccine is shown as the proportion of patients with 90% confidence interval (CI) with response detected at d183 (of those evaluated ex vivo (**c**) and after IVS (**d**). Also for group 23 (pICLC, V6), the measured immune response magnitudes are shown through week 26 ex vivo (**e**) and IVS (**f**)

Durability of CD8 T cell responses was assessed by number of time points with positive responses to 12MP after start of vaccine treatment (weeks 1 or later) and by the percent of participants evaluated who had T cell responses at week 26 (wk26). Median numbers of time points with ex vivo responses to 12MP, for V0, V1, V6, respectively, were 0, 0, and 1.5 for LPS and 0, 1.5, and 2.5 for polyICLC. For IVS assays, those values were 0, 0.5, and 4, for LPS, and 1.5, 2, and 4 for polyICLC (Fig. 4a, b), representing significant increases overall from V0 to V6 (LR *p* = 0.022 and *p* < 0.001 for ex vivo and IVS, respectively) but not for V0 to V1 (LR *p* = 0.4 and *p* = 0.3 for ex vivo and IVS, respectively).

Persistence of T cell responses in PBMC at wk26 was evaluable by ex vivo ELIspot (*n* = 30) and in IVS ELIspot (*n* = 40) assays. At this late time point, CD8 T cell responses to 12MP were detected ex vivo in 13%, and after IVS in 48%. Ex vivo responses at wk26 were detected only in cohort 2 patients who had IFA included (V1 and V6) (Fig. 4c). After IVS, responses were detected wk26 in 14, 42, and 86% of patients in V0, V1, and V6 subgroups, respectively (*n* = 14, 12, 14, respectively) (Fig. 4d). The increase for V6 versus V1 versus V0 overall was significant for IVS assay results only (LR p < 0.001) Thus, persistent responses were significantly enhanced with inclusion of IFA in all 6 vaccines, compared to use of TLR agonists alone, and were similar with either TLR agonist, though they may be slightly more common with polyICLC than with LPS.

### Immune response rates summarized by HLA type

Patients expressing HLA-A1, A2, A3, or other A3 supertype alleles (A11, A31), were represented in each cohort, and CD8^+^ T cell responses were identified among patients expressing each HLA subtype (Additional file 1: Tables S6 and S7). There were differences in immunogenicity among the individual peptides, as previously observed [7, 8, 28]. By IVS ELIspot, the highest response rates were to the HLA-A2 peptide IMD (gp100_209–217 (2M)_) (68%), HLA-A1 peptide DAE (tyrosinase_240-251S_) (59%), HLA-A3 peptide SLF (MAGE-A1_96–104_) (43%), and the HLA-A2 peptide GLY (MAGE-A10_254–262_) (52%) (Additional file 1: Table S8). For 9/12 peptides, the immune response rates were higher in Cohort 2 than in Cohort 1, and for 2 of them the immune response rates were 0 in both; only one peptide (YMD) had an immune response rate marginally higher in Cohort 1 (29% vs 25%). No apparent differences in durability of immune response were observed among different HLA alleles (Additional file 1: Table S9).

### Clinical outcome

Overall survival and disease-free survival were high for the entire study population. The study was not powered to investigate changes in overall and disease-free survival among study groups, but they appear similar thus far. (Additional file 1: Figure S3).

## Discussion

There is no consensus on best adjuvants to support strong and durable T cell responses to cancer antigens. Our prior work has demonstrated that vaccines using peptides emulsified in IFA can induce CD8 T cell responses in 70–80% of patients based on ex vivo IVS ELIspot assays, and can also induce CD4 T cell responses in most patients, while also supporting induction of peptide-specific antibody responses [31]. The immune responses can exceed 5% of circulating CD8 T cells after vaccination with peptides in IFA alone [1, 28]. However, some T cell responses with IFA are transient and not all patients develop strong responses [4]. Thus, there is interest in enhancing T cell responses to vaccines.

Concerns about use of IFA have been raised by murine studies, which showed that peptide vaccination in IFA induced inflammation at vaccine sites that selectively recruited and depleted peptide-specific T cells, thereby negatively impacting tumor control [5, 6]. Multiple investigators have induced strong and durable CD8 T cell responses to short peptides in mice using adjuvants combining a TLR agonist and an agonistic CD40 Ab [5, 32, 33]; however, this approach has not yet been evaluated in humans. The Mel58 clinical trial was designed to test whether vaccination with minimal epitope melanoma peptides in a TLR agonist, combined with helper T cell activation, would be more effective at inducing durable T cell responses than use of the same adjuvant preparation combined with IFA. However, in contrast to our underlying hypothesis, we found that circulating CD8 T cell responses to minimal epitopes were greater in magnitude and durability when IFA was included, especially when IFA was included in all 6 vaccines.

The trial tested agonists for both TLR3 and TLR4. TLR4 agonists have also been studied as vaccine adjuvants, but the classic TLR4 agonist, LPS, has long been considered too toxic for human use. However, the present formulation of GMP grade endotoxin has a strong safety profile [34–38]. Human experience with it, administered systemically, either by intravenous injection or by inhalation, is that it causes systemic inflammatory responses that are transient and very well-tolerated up to 2500 EU per dose [34, 35, 39]. LPS is known to activate innate immunity, however; to our knowledge, it has not been previously been used as a vaccine adjuvant. In the present study, we escalated from 25 EU to 1600 EU, with and without IFA, and there were no DLTs. Thus, these data support the safety of bacterial LPS as a vaccine adjuvant. Interestingly, one patient had skin hypopigmentation (patient 27, LPS 1600, V0) though no T cell response to 12MP or tetanus peptide ex vivo, but positive to tyrosinase (DAEK) with in vitro stimulated ELIspot assay in PBMC and SIN (Additional file 1: Table S4). The goal of the rapid dose-escalation was to define safety at the maximal tolerated dose, up to 1600 EU. Since that dose was found safe, most patients in Cohort 1 were enrolled at 1600 EU dose, limiting the ability to determine which of the 4 LPS doses is most immunogenic. Within this constraint, no significant difference in immunogenicity was observed among the LPS doses.

The TLR3 agonist polyICLC has been studied in preclinical models and in clinical trials [40–42], with favorable safety and immunogenicity profiles, and, when combined with IFA, it has been shown to enhance CD4, CD8, and antibody responses to long NY-ESO-1 peptides compared to IFA alone [3]. Sabbatini et al. did observe marked injection site reactions in 2 patients treated with NYESO1 long peptides plus IFA and 1.4 mg polyICLC, and discontinued treatment early for 4 of 11 patients. Considering this, we employed a lower dose (1 mg) in our study [3]. We observed injection site reactions that met stopping criteria for 1 of 6 patients in arm 22 (polyICLC, V1) and that contributed to the overall DLT for one of 6 patients in arm 23 (polyICLC, V6). However, these were not serious adverse events, which resolved after stopping treatment. These DLTs did not meet stopping criteria for any sub-arm of cohort 2. Thus, the regimen is considered safe; however, prominent local injection site reactions can be expected. Overall, the data support polyICLC as an effective vaccine adjuvant when combined with IFA, for inducing CD8 T cell responses to minimal peptides, with an acceptable safety profile. This regimen appears marginally better than LPS plus IFA, which was very well-tolerated, but also supported immune responses. Other TLR agonists have been shown to enhance T cell responses to peptides in vaccines, in particular TLR9 agonist CpG-B (7909, PF-3512676) [43, 44]. Thus, a range of TLR agonists have value in combination with IFA as vaccine adjuvants.

For an adjuvant to have maximum benefit, it has to generate an antigen depot, to activate APC, and to provide co-stimulation through CD4 T cell help [45]. The present study provided an antigen depot with the water-in-oil emulsion with IFA, TLR agonists to activate APC, and a tetanus peptide that is very effective at inducing CD4 helper T cell responses. Activation of CD4 helper T cells will induce CD40L expression, which in turn can license APC and enhance their antigen presentation. We have not formally tested the impact of CD40L expression by tetanus-reactive CD4 T cells but have found in this trial that T cell responses to the tetanus helper peptide was greater with inclusion of IFA (V1 or V6) than without it (V0). Thus, the impact of IFA may include both a direct effect on the CD8 T cell response and an indirect effect, through activation of CD4 T cells, and subsequent APC activation.

In the murine studies that have shown negative effects of IFA on CD8 T cell responses to short peptides, an alternative vaccination approach using a water-soluble adjuvant preparation, including TLR7 agonist imiquimod and CD40 antibody induced more durable immune responses and better tumor control [5]. Also, strong CD8 responses have been induced in mice by co-administration of peptides, CD40 Ab, and PolyIC [46]. Clinical grade human agonistic antibodies to CD40 were not available at the time of the present clinical trial. Thus, the vaccine regimen included the tetanus helper peptide to enhance CD4 help via CD40L expression. It will be valuable to reconcile the favorable findings from this trial in light of the unfavorable findings with use of IFA in murine models. There are several differences in the experimental setting for the murine studies and that of the present clinical trial. These include dose and volume differences in the vaccine, differences in T cell frequency, inclusion of helper peptide in the human trial, as well as potential differences between human and mouse. In the murine studies, 100 mcg of peptide was given in a 100 mcl emulsion with IFA [5, 6]. In the human trial, 100 mcg of each peptide was given in a 1 ml emulsion with IFA. Considering the impact of the large depot in the mouse, volume of that depot is likely relevant to the observed findings. A mouse typically weighs about 25 g; thus, 100 mcl represents 1/250th of the mass of the mouse. For a 70 kg human, the 1 ml emulsion used in the clinical trial represents 1/70,000th of the mass of the patient. This 280-fold v/v difference is dramatic: If the patients had been administered a 280 ml emulsion, a much more dramatic vaccine site effect might be anticipated. Also, the murine studies used adoptive transfer of 1 × 10^6^ activated antigen-reactive T cells which represents about 50% of circulating CD8 T cells [47]. This exceeds the pre-treatment frequency of antigen-specific T cells in humans, probably by at least 2 logs, and also exceeds what is induced over time with vaccination. Thus, the administration of a high dose of antigen-reactive T cells into a massive IFA depot may explain in part the difference between the experimental findings in the mouse and what is observed in this clinical trial. Also, this trial included T cell help, in the form of a tetanus helper peptide, which was not included in the murine studies. We have found that vaccination with IFA plus TLR agonist and inclusion of CD4 help induced a high rate of T cell responses ex vivo, durable in most patients for at least 6 months.

In prior work, we observed transient responses by circulating T cells [4, 28] and that T cells accumulate at sites of vaccination with peptides in IFA [48, 49]. These observations, in light of murine data on IFA as an adjuvant [5], suggested a decline of responsive circulating T cells due to accumulation at vaccine sites.. Alternatively, the transient responses observed with direct ELIspots may be explained by reversion of effector T cells to memory, especially after the vaccine sequence is completed. As such, they may not be detected as effectors ex vivo but are functional after restimulation. In support of this, we observed stimulated responses out to wk. 26 in 85% of the evaluable patients with V6 IFA (Fig. 2b), compared to 50 and 14% respectively with V1 and V0. Therefore repeated doses of IFA may support durable memory responses rather than accumulation and depletion at vaccine sites.

New strategies for vaccination against mutated neoantigens have promise for enhancing immune repertoires; however, the clinical trials of neoantigen vaccines published to date have all used different vaccine adjuvant strategies, and most of the T cell responses induced were detectable only after in vitro stimulation [50–52]. Thus, enhanced strategies for vaccination remain a high priority for the field. The present study suggests that a TLR agonist alone may not be sufficient for induction of a strong T cell response to a peptide vaccine, and that inclusion of IFA with a helper peptide remains an effective strategy. Future trials should test whether addition of a CD40 antibody plus TLR agonist at the vaccine site can further enhance T cell responses in patients, with or without IFA.

## Conclusions

This clinical trial was designed to test whether vaccination with 12 short melanoma peptides in combination with TLR agonists polyICLC or LPS with IFA was safe and immunogenic in melanoma patients. Only 2 DLTs were observed, in different sub-arms of cohort 2 (polyICLC): no treatment combination met stopping criteria. A driving hypothesis was that inclusion of IFA with TLR agonists would be less effective in generating a durable T cell response. However, in contrast to our hypothesis, peptide-specific CD8 T cell responses were more durable and of greater magnitude when IFA was included as an adjuvant, regardless of whether it was combined with polyICLC or LPS. Furthermore, our study suggests that, overall, polyICLC may induce marginally better CD8 T cell responses than LPS. Future studies will aim to understand mechanisms underlying the favorable effects with IFA.

## Additional files

Additional file 1: **Table S1**. Peptides used in vaccines. **Table S2**. Target Study Groups and Subgroups. **Table S3**. Patient demographics. **Table S4**. Treatment-related adverse events. **Table S5**. Direct ELIspot: number of patients in each study group with T cell response through week 26. **Table S6**. Patient information, treatment regimen and ELIspot responses summarized per patient. **Table S7**. HLA expression by study group and subgroup, and associated immune response rates through week 26. **Table S8**. Stimulated ELIspot response rate by cohort (N and %) through week 26. **Table S9**. T cell response by IVS ELIspot to 12MP at day 183, among evaluable patients, by HLA type. **Figure S1**. Immune responses in patients with DLTs. **Figure S2**. T cell responses to tetanus peptide, measured by direct ELIspot assay. **Figure S3.** Survival and progression free survival curves, comparing cohort 1 and 2 (A) and IFA groups V0, V1 and V6 (B). (DOCX 1332 kb)

Additional file 2: Supplemental Text. (DOCX 38 kb)

## Data Availability

The datasets used and/or analyzed during the current study are available from the corresponding author on reasonable request.

## Abbreviations

12MP: 12 melanoma peptides
APC: Antigen-presenting cell
CV: Coefficient of variation
DLT: Dose-limiting toxicity
EU: Endotoxin Unit
IFA: Incomplete Freund’s Adjuvant
IVS: In vitro stimulation
LPS: Lipopolysaccharide
LR: Likelihood of chi-square test statistic
MTDC: Maximum tolerated dose combination
PS: Performance status
SIN: Sentinel immunized node
Tet: Tetanus helper peptide
TLR: Toll-like receptor
V0: No IFA
V1: IFA with first vaccine
V6: IFA with all six vaccines

## References

[1] Rosenberg SA, Sherry RM, Morton KE, Scharfman WJ, Yang JC, Topalian SL, Royal RE, Kammula U, Restifo NP, Hughes MS (2005). Tumor progression can occur despite the induction of very high levels of self/tumor antigen-specific CD8+ T cells in patients with melanoma. J Immunol.

[2] Obeid J, Hu Y, Slingluff CL (2015). Vaccines, adjuvants, and dendritic cell activators--current status and future challenges. Semin Oncol.

[3] Sabbatini P, Tsuji T, Ferran L, Ritter E, Sedrak C, Tuballes K, Jungbluth AA, Ritter G, Aghajanian C, Bell-McGuinn K (2012). Phase I trial of overlapping long peptides from a tumor self-antigen and poly-ICLC shows rapid induction of integrated immune response in ovarian cancer patients. Clin Cancer Res.

[4] Slingluff CL, Petroni GR, Yamshchikov GV, Hibbitts S, Grosh WW, Chianese-Bullock KA, Bissonette EA, Barnd DL, Deacon DH, Patterson JW (2004). Immunologic and clinical outcomes of vaccination with a multiepitope melanoma peptide vaccine plus low-dose interleukin-2 administered either concurrently or on a delayed schedule. J Clin Oncol.

[5] Hailemichael Y, Dai Z, Jaffarzad N, Ye Y, Medina MA, Huang XF, Dorta-Estremera SM, Greeley NR, Nitti G, Peng W (2013). Persistent antigen at vaccination sites induces tumor-specific CD8(+) T cell sequestration, dysfunction and deletion. Nat Med.

[6] Hailemichael Yared, Woods Amber, Fu Tihui, He Qiuming, Nielsen Michael C., Hasan Farah, Roszik Jason, Xiao Zhilan, Vianden Christina, Khong Hiep, Singh Manisha, Sharma Meenu, Faak Faisal, Moore Derek, Dai Zhimin, Anthony Scott M., Schluns Kimberly S., Sharma Padmanee, Engelhard Victor H., Overwijk Willem W. (2018). Cancer vaccine formulation dictates synergy with CTLA-4 and PD-L1 checkpoint blockade therapy. Journal of Clinical Investigation.

[7] Slingluff CL, Petroni GR, Chianese-Bullock KA, Smolkin ME, Hibbitts S, Murphy C, Johansen N, Grosh WW, Yamshchikov GV, Neese PY (2007). Immunologic and clinical outcomes of a randomized phase II trial of two multipeptide vaccines for melanoma in the adjuvant setting. Clin Cancer Res.

[8] Slingluff CL, Petroni GR, Chianese-Bullock KA, Smolkin ME, Ross MI, Haas NB, von Mehren M, Grosh WW (2011). Randomized multicenter trial of the effects of melanoma-associated helper peptides and cyclophosphamide on the immunogenicity of a multipeptide melanoma vaccine. J Clin Oncol.

[9] van Mierlo GJ, den Boer AT, Medema JP, van der Voort EI, Fransen MF, Offringa R, Melief CJ, Toes RE (2002). CD40 stimulation leads to effective therapy of CD40(−) tumors through induction of strong systemic cytotoxic T lymphocyte immunity. Proc Natl Acad Sci U S A.

[10] Schoenberger SP, Toes RE, van der Voort EI, Offringa R, Melief CJ (1998). T-cell help for cytotoxic T lymphocytes is mediated by CD40-CD40L interactions. Nature.

[11] Melssen M, Slingluff CL (2017). Vaccines targeting helper T cells for cancer immunotherapy. Curr Opin Immunol.

[12] Slingluff CL, Yamshchikov G, Neese P, Galavotti H, Eastham S, Engelhard VH, Kittlesen D, Deacon D, Hibbitts S, Grosh WW (2001). Phase I trial of a melanoma vaccine with gp100(280-288) peptide and tetanus helper peptide in adjuvant: immunologic and clinical outcomes. Clin Cancer Res.

[13] Slingluff CL, Yamshchikov GV, Hogan KT, Hibbitts SC, Petroni GR, Bissonette EA, Patterson JW, Neese PY, Grosh WW, Chianese-Bullock KA (2008). Evaluation of the sentinel immunized node for immune monitoring of cancer vaccines. Ann Surg Oncol.

[14] Wages NA, Conaway MR, O'Quigley J (2011). Dose-finding design for multi-drug combinations. Clin Trials.

[15] O'Quigley J, Pepe M, Fisher L (1990). Continual reassessment method: a practical design for phase 1 clinical trials in cancer. Biometrics.

[16] Cox AL, Skipper J, Chen Y, Henderson RA, Darrow TL, Shabanowitz J, Engelhard VH, Hunt DF, Slingluff CL (1994). Identification of a peptide recognized by five melanoma-specific human cytotoxic T cell lines. Science.

[17] Kittlesen DJ, Thompson LW, Gulden PH, Skipper JC, Colella TA, Shabanowitz J, Hunt DF, Engelhard VH, Slingluff CL (1998). Human melanoma patients recognize an HLA-A1-restricted CTL epitope from tyrosinase containing two cysteine residues: implications for tumor vaccine development [published erratum appears in J Immunol 1999 Mar 1;162(5):3106]. J Immunol.

[18] Skipper JC, Hendrickson RC, Gulden PH, Brichard V, Van Pel A, Chen Y, Shabanowitz J, Wolfel T, Slingluff CL, Boon T (1996). An HLA-A2-restricted tyrosinase antigen on melanoma cells results from posttranslational modification and suggests a novel pathway for processing of membrane proteins. J Exp Med.

[19] Skipper JC, Kittlesen DJ, Hendrickson RC, Deacon DD, Harthun NL, Wagner SN, Hunt DF, Engelhard VH, Slingluff CL (1996). Shared epitopes for HLA-A3-restricted melanoma-reactive human CTL include a naturally processed epitope from Pmel-17/gp100. J Immunol.

[20] Chaux P, Luiten R, Demotte N, Vantomme V, Stroobant V, Traversari C, Russo V, Schultz E, Cornelis GR, Boon T, van der Bruggen P (1999). Identification of five MAGE-A1 epitopes recognized by cytolytic T lymphocytes obtained by in vitro stimulation with dendritic cells transduced with MAGE-A1. J Immunol.

[21] Kawakami Y, Robbins PF, Wang X, Tupesis JP, Parkhurst MR, Kang X, Sakaguchi K, Appella E, Rosenberg SA (1998). Identification of new melanoma epitopes on melanosomal proteins recognized by tumor infiltrating T lymphocytes restricted by HLA-A1, −A2, and -A3 alleles. J Immunol.

[22] Wang RF, Johnston SL, Zeng G, Topalian SL, Schwartzentruber DJ, Rosenberg SA (1998). A breast and melanoma-shared tumor antigen: T cell responses to antigenic peptides translated from different open reading frames. J Immunol.

[23] Huang LQ, Brasseur F, Serrano A, De Plaen E, van der Bruggen P, Boon T, Van Pel A (1999). Cytolytic T lymphocytes recognize an antigen encoded by MAGE-A10 on a human melanoma. J Immunol.

[24] Traversari C, van der Bruggen P, Luescher IF, Lurquin C, Chomez P, Van Pel A, De Plaen E, Amar-Costesec A, Boon T (1992). A nonapeptide encoded by human gene MAGE-1 is recognized on HLA-A1 by cytolytic T lymphocytes directed against tumor antigen MZ2-E. J Exp Med.

[25] Kawakami Y, Eliyahu S, Jennings C, Sakaguchi K, Kang X, Southwood S, Robbins PF, Sette A, Appella E, Rosenberg SA (1995). Recognition of multiple epitopes in the human melanoma antigen gp100 by tumor-infiltrating T lymphocytes associated with in vivo tumor regression. J Immunol.

[26] Gaugler B, Van den Eynde B, van der Bruggen P, Romero P, Gaforio JJ, De Plaen E, Lethe B, Brasseur F, Boon T (1994). Human gene MAGE-3 codes for an antigen recognized on a melanoma by autologous cytolytic T lymphocytes. J Exp Med.

[27] Panina-Bordignon P, Tan A, Termijtelen A, Demotz S, Corradin G, Lanzavecchia A (1989). Universally immunogenic T cell epitopes: promiscuous binding to human MHC class II and promiscuous recognition by T cells. Eur J Immunol.

[28] Slingluff CL, Petroni GR, Olson WC, Smolkin ME, Ross MI, Haas NB, Grosh WW, Boisvert ME, Kirkwood JM, Chianese-Bullock KA (2009). Effect of granulocyte/macrophage colony-stimulating factor on circulating CD8+ and CD4+ T-cell responses to a multipeptide melanoma vaccine: outcome of a multicenter randomized trial. Clin Cancer Res.

[29] Slingluff CL, Lee S, Zhao F, Chianese-Bullock KA, Olson WC, Butterfield LH, Whiteside TL, Leming PD, Kirkwood JM (2013). A randomized phase II trial of multiepitope vaccination with melanoma peptides for cytotoxic T cells and helper T cells for patients with metastatic melanoma (E1602). Clin Cancer Res.

[30] Good PI (2005). Permutation, parametric, and bootstrap tests of hypotheses.

[31] Reed CM, Cresce ND, Mauldin IS, Slingluff CL, Olson WC (2015). Vaccination with melanoma helper peptides induces antibody responses associated with improved overall survival. Clin Cancer Res.

[32] Cho HI, Jung SH, Sohn HJ, Celis E, Kim TG (2015). An optimized peptide vaccine strategy capable of inducing multivalent CD8(+) T cell responses with potent antitumor effects. Oncoimmunology.

[33] Sanchez PJ, McWilliams JA, Haluszczak C, Yagita H, Kedl RM (2007). Combined TLR/CD40 stimulation mediates potent cellular immunity by regulating dendritic cell expression of CD70 in vivo. J Immunol.

[34] Talwar S, Munson PJ, Barb J, Fiuza C, Cintron AP, Logun C, Tropea M, Khan S, Reda D, Shelhamer JH (2006). Gene expression profiles of peripheral blood leukocytes after endotoxin challenge in humans. Physiol Genomics.

[35] Preas HL, Jubran A, Vandivier RW, Reda D, Godin PJ, Banks SM, Tobin MJ, Suffredini AF (2001). Effect of endotoxin on ventilation and breath variability: role of cyclooxygenase pathway. Am J Respir Crit Care Med.

[36] O'Grady NP, Preas HL, Pugin J, Fiuza C, Tropea M, Reda D, Banks SM, Suffredini AF (2001). Local inflammatory responses following bronchial endotoxin instillation in humans. Am J Respir Crit Care Med.

[37] Bornstein SR, Wolkersdorfer GW, Tauchnitz R, Preas HL, Chrousos GP, Suffredini AF (2000). Plasma dehydroepiandrosterone levels during experimental endotoxemia and anti-inflammatory therapy in humans. Crit Care Med.

[38] Suffredini AF, Hochstein HD, McMahon FG (1999). Dose-related inflammatory effects of intravenous endotoxin in humans: evaluation of a new clinical lot of Escherichia coli O:113 endotoxin. J Infect Dis.

[39] Preas HL, Nylen ES, Snider RH, Becker KL, White JC, Agosti JM, Suffredini AF (2001). Effects of anti-inflammatory agents on serum levels of calcitonin precursors during human experimental endotoxemia. J Infect Dis.

[40] Mehrotra S, Britten CD, Chin S, Garrett-Mayer E, Cloud CA, Li M, Scurti G, Salem ML, Nelson MH, Thomas MB (2017). Vaccination with poly (IC:LC) and peptide-pulsed autologous dendritic cells in patients with pancreatic cancer. J Hematol Oncol.

[41] Tewari Kavita, Flynn Barbara J., Boscardin Silvia B., Kastenmueller Kathrin, Salazar Andres M., Anderson Charles A., Soundarapandian Velmurugan, Ahumada Adriana, Keler Tibor, Hoffman Stephen L., Nussenzweig Michel C., Steinman Ralph M., Seder Robert A. (2010). Poly(I:C) is an effective adjuvant for antibody and multi-functional CD4+ T cell responses to Plasmodium falciparum circumsporozoite protein (CSP) and αDEC-CSP in non human primates. Vaccine.

[42] Zhu X, Nishimura F, Sasaki K, Fujita M, Dusak JE, Eguchi J, Fellows-Mayle W, Storkus WJ, Walker PR, Salazar AM, Okada H (2007). Toll like receptor-3 ligand poly-ICLC promotes the efficacy of peripheral vaccinations with tumor antigen-derived peptide epitopes in murine CNS tumor models. J Transl Med.

[43] Baumgaertner P, Costa Nunes C, Cachot A, Maby-El Hajjami H, Cagnon L, Braun M, Derre L, Rivals JP, Rimoldi D, Gnjatic S (2016). Vaccination of stage III/IV melanoma patients with long NY-ESO-1 peptide and CpG-B elicits robust CD8+ and CD4+ T-cell responses with multiple specificities including a novel DR7-restricted epitope. Oncoimmunology.

[44] Speiser DE, Lienard D, Rufer N, Rubio-Godoy V, Rimoldi D, Lejeune F, Krieg AM, Cerottini JC, Romero P (2005). Rapid and strong human CD8+ T cell responses to vaccination with peptide, IFA, and CpG oligodeoxynucleotide 7909. J Clin Investig.

[45] Overwijk WW (2017). Cancer vaccines in the era of checkpoint blockade: the magic is in the adjuvant. Curr Opin Immunol.

[46] Cho HI, Celis E (2009). Optimized peptide vaccines eliciting extensive CD8 T-cell responses with therapeutic antitumor effects. Cancer Res.

[47] Pinchuk LM, Filipov NM (2008). Differential effects of age on circulating and splenic leukocyte populations in C57BL/6 and BALB/c male mice. Immun Ageing.

[48] Salerno EP, Shea SM, Olson WC, Petroni GR, Smolkin ME, McSkimming C, Chianese-Bullock KA, Slingluff CL (2013). Activation, dysfunction and retention of T cells in vaccine sites after injection of incomplete Freund's adjuvant, with or without peptide. Cancer Immunol Immunother.

[49] Schaefer JT, Patterson JW, Deacon DH, Smolkin ME, Petroni GR, Jackson EM, Slingluff CL (2010). Dynamic changes in cellular infiltrates with repeated cutaneous vaccination: a histologic and immunophenotypic analysis. J Transl Med.

[50] Ott Patrick A., Hu Zhuting, Keskin Derin B., Shukla Sachet A., Sun Jing, Bozym David J., Zhang Wandi, Luoma Adrienne, Giobbie-Hurder Anita, Peter Lauren, Chen Christina, Olive Oriol, Carter Todd A., Li Shuqiang, Lieb David J., Eisenhaure Thomas, Gjini Evisa, Stevens Jonathan, Lane William J., Javeri Indu, Nellaiappan Kaliappanadar, Salazar Andres M., Daley Heather, Seaman Michael, Buchbinder Elizabeth I., Yoon Charles H., Harden Maegan, Lennon Niall, Gabriel Stacey, Rodig Scott J., Barouch Dan H., Aster Jon C., Getz Gad, Wucherpfennig Kai, Neuberg Donna, Ritz Jerome, Lander Eric S., Fritsch Edward F., Hacohen Nir, Wu Catherine J. (2017). An immunogenic personal neoantigen vaccine for patients with melanoma. Nature.

[51] Sahin U, Derhovanessian E, Miller M, Kloke BP, Simon P, Lower M, Bukur V, Tadmor AD, Luxemburger U, Schrors B, et al. Personalized RNA mutanome vaccines mobilize poly-specific therapeutic immunity against cancer. Nature. 2017;547(7662):222-6. 10.1038/nature23003 28678784

[52] Carreno BM, Magrini V, Becker-Hapak M, Kaabinejadian S, Hundal J, Petti AA, Ly A, Lie WR, Hildebrand WH, Mardis ER, Linette GP (2015). Cancer immunotherapy. A dendritic cell vaccine increases the breadth and diversity of melanoma neoantigen-specific T cells. Science.

