# Supplementary material for: A multipeptide vaccine plus toll-like receptor agonists LPS or polyICLC in combination with incomplete Freund’s adjuvant in melanoma patients
Source: J Immunother Cancer. 2019 Jun 27;7:163. doi: 10.1186/s40425-019-0625-x (PMC6598303; doi:10.1186/s40425-019-0625-x)
Supplement: Supplementary file 1 — Table S1. Peptides used in vaccines. Table S2. Target Study Groups and Subgroups. Table S3. Patient demographics. Table S4. Treatment-related adverse events. Table S5. Direct ELIspot: number of patients in each study group with T cell response through week 26. Table S6. Patient information, treatment regimen and ELIspot responses summarized per patient. Table S7. HLA expression by study group and subgroup, and associated immune response rates through week 26. Table S8. Stimulated ELIspot response rate by cohort (N and %) through week 26. Table S9. T cell response by IVS ELIspot to 12MP at day 183, among evaluable patients, by HLA type. Figure S1. Immune responses in patients with DLTs. Figure S2. T cell responses to tetanus peptide, measured by direct ELIspot assay. Figure S3. Survival and progression free survival curves, comparing cohort 1 and 2 (A) and IFA groups V0, V1 and V6 (B). (DOCX 1332 kb) [file 40425_2019_625_MOESM1_ESM.docx]

**Supplemental Table 1. Peptides used in vaccines**

| Prep | Allele | | Sequence | Epitope |
| --- | --- | --- | --- | --- |
| 12MP | HLA-A1 | DAEKSDICTDEY | | Tyrosinase _240-251_ * |
|  |  | SSDYVIPIGTY | | Tyrosinase _146-156_ |
|  |  | EADPTGHSY | | MAGE-A1 _161-169_ |
|  |  | EVDPIGHLY | | MAGE-A3 _168-176_ |
|  | HLA-A2 | YMDGTMSQV | | Tyrosinase _369-377_ ♦ |
|  |  | IMDQVPFSV | | gp100 _209-217_ # |
|  |  | YLEPGPVTA | | gp100 _280-288_ |
|  |  | GLYDGMEHL | | MAGE-A10 _254-262_ |
|  | HLA-A3/A11/A31 | ALLAVGATK | | gp100 _17-25_ |
|  |  | LIYRRRLMK | | gp100 _614-622_ |
|  |  | SLFRAVITK | | MAGE-A1 _96-104_ |
|  |  | ASGPGGGAPR | | NY-ESO-1 _53-62_ |
| Tet | HLA-DR (multiple) | AQYIKANSKFIGITEL | | Tetanus toxoid p2_830-844_** |

* substitution of S for C, at residue 244.

♦(post-translational change of N to D at residue 371)

#(209-2M, substitution of M for T at residue 210)

**An alanine residue was added to the N-terminus to prevent cyclization.

| **Supplemental Table 2. Target Study Groups and Subgroups** | | | | | |
| --- | --- | --- | --- | --- | --- |
| Study  cohort | Peptide vaccine | TLR agonist | Dose of TLR  agonist | IFA | Route peptide  + TLR agonist |
| 1 | MELITAC 12.1 (12MP + Tet) | LPS* | Escalation 25,  100, 400, 1600 EU | 1. None (0) | Id/sq |
|  |  |  |  | 1. Vaccine 1 (V1) | Id/sq |
|  |  |  |  | 1. All vaccines (V6) | Id/sq |
| 2 | MELITAC 12.1 (12MP + Tet) | polyICLC | 1 mg | 1. None (0) | Id/sq |
|  |  |  |  | 1. Vaccine 1 (V1) | Id/sq |
|  |  |  |  | c) All vaccines (V6) | Id/sq |
| * LPS = lipopolysaccharide (endotoxin) | | | | | |

**Supplemental Table 3. Patient demographics**

|  | **Arm 1.**  **LPS** | **Arm 2.**  **pICLC** | **Overall** |
| --- | --- | --- | --- |
| N | 33 | 18 | 51 |
| Race |  |  |  |
| White | 33 | 17 | 50 |
| Asian | 0 | 1 | 1 |
|  |  |  |  |
| Gender |  |  |  |
| F | 7 | 12 | 19 |
| M | 26 | 6 | 32 |
| Ethnicity |  |  |  |
| Hispanic | 2 | 0 | 2 |
|  |  |  |  |
| ECOG PS at registration |  |  |  |
| 0 | 28 | 18 | 46 |
| 1 | 5 | 0 | 5 |
|  |  |  |  |
| HLA* |  |  |  |
| A1+ | 15 | 7 | 22 |
| A2+ | 17 | 8 | 25 |
| A3+ | 8 | 6 | 14 |
| A11/31 | 5 | 4 | 9 |
|  |  |  |  |
| Primary site |  |  |  |
| Skin, non-acral | 26 | 17 | 43 |
| Unknown | 4 | 0 | 4 |
| Acral | 2 | 1 | 3 |
| Ocular | 1 | 0 | 1 |
|  |  |  |  |
| Stage at Registration** |  |  |  |
| IIB-IIC | 4 | 1 | 5 |
| III | 25 | 15 | 40 |
| IIIA | 6 | 2 | 8 |
| IIIB/C | 19 | 13 | 32 |
| IV | 4 | 2 | 6 |
|  |  |  |  |
| LDH |  |  |  |
| <=ULN | 31 | 17 | 48 |
| >ULN | 1 | 1 | 2 |
| Missing | 1 | 0 | 1 |

**Supplemental Table 4. Treatment-related adverse events**

| **MEL 58 Maximum Grade Toxicities (Related)** | | | | | | | | | | |
| --- | --- | --- | --- | --- | --- | --- | --- | --- | --- | --- |
|  | | Cohort Totals | | | | | |  | | |
|  | | LPS | | | PolyICLC | | | Total | | |
| Toxicity Category | Toxicity Description | G1 | G2 | G3 | G1 | G2 | G3 | G1 | G2 | G3 |
| BLOOD AND LYMPHATIC SYSTEM DISORDERS | ANEMIA | 1 |  |  | 1 |  |  | 2 |  |  |
| EAR AND LABYRINTH DISORDERS | TINNITUS | 1 |  |  |  |  |  | 1 |  |  |
| EYE DISORDERS | OTHER | 1 |  |  |  |  |  | 1 |  |  |
| GASTROINTESTINAL DISORDERS | DIARRHEA | 2 |  |  |  | 1 |  | 2 | 1 |  |
|  | DRY MOUTH |  |  |  | 1 |  |  | 1 |  |  |
|  | MUCOSITIS ORAL | 1 |  |  |  | 1 |  | 1 | 1 |  |
|  | NAUSEA | 3 |  |  | 3 |  |  | 6 |  |  |
|  | VOMITING |  |  |  | 1 |  |  | 1 |  |  |
| GENERAL DISORDERS AND ADMINISTRATION SITE CONDITIONS | CHILLS | 5 |  |  | 2 | 2 |  | 7 | 2 |  |
|  | EDEMA LIMBS |  |  |  |  | 1 |  |  | 1 |  |
|  | FATIGUE | 14 | 3 |  | 12 | 2 |  | 26 | 5 |  |
|  | FEVER | 4 |  |  | 3 |  |  | 7 |  |  |
|  | FLU LIKE SYMPTOMS | 4 | 2 |  | 3 | 1 |  | 7 | 3 |  |
|  | INJECTION SITE REACTION | 16 | 15 |  | 2 | 16 |  | 18 | 31 |  |
|  | PAIN | 2 |  |  |  |  |  | 2 |  |  |
| IMMUNE SYSTEM DISORDERS | AUTOIMMUNE DISORDER | 2 |  |  | 2 |  |  | 4 |  |  |
|  | CYTOKINE RELEASE SYNDROME |  |  |  |  | 1 |  |  | 1 |  |
| INJURY, POISONING AND PROCEDURAL COMPLICATIONS | SEROMA | 2 |  |  |  |  |  | 2 |  |  |
|  | WOUND DEHISCENCE | 2 |  |  |  |  |  | 2 |  |  |
| INVESTIGATIONS | LYMPHOCYTE COUNT DECREASED |  |  |  |  | 2 |  |  | 2 |  |
|  | WEIGHT GAIN |  |  |  | 1 |  |  | 1 |  |  |
|  | WHITE BLOOD CELL DECREASED | 1 |  |  | 1 | 1 |  | 2 | 1 |  |
| METABOLISM AND NUTRITION DISORDERS | ANOREXIA | 2 |  |  | 1 |  |  | 3 |  |  |
|  | DEHYDRATION |  |  |  | 1 |  |  | 1 |  |  |
| MUSCULOSKELETAL AND CONNECTIVE TISSUE DISORDERS | ARTHRALGIA | 10 | 1 |  | 5 |  |  | 15 | 1 |  |
|  | MYALGIA | 5 |  |  | 5 |  |  | 10 |  |  |
|  | PAIN IN EXTREMITY | 1 |  |  |  |  |  | 1 |  |  |
| NERVOUS SYSTEM DISORDERS | CONCENTRATION IMPAIRMENT | 1 |  |  |  |  |  | 1 |  |  |
|  | DIZZINESS | 2 |  |  |  |  |  | 2 |  |  |
|  | HEADACHE | 4 |  |  | 5 | 1 |  | 9 | 1 |  |
| PSYCHIATRIC DISORDERS | AGITATION |  |  |  | 1 |  |  | 1 |  |  |
|  | ANXIETY | 1 |  |  |  |  |  | 1 |  |  |
| RESPIRATORY, THORACIC AND MEDIASTINAL DISORDERS | ALLERGIC RHINITIS | 2 |  |  |  |  |  | 2 |  |  |
|  | COUGH |  |  |  | 1 |  |  | 1 |  |  |
|  | DYSPNEA | 1 |  |  |  |  |  | 1 |  |  |
|  | NASAL CONGESTION | 1 |  |  |  |  |  | 1 |  |  |
|  | SORE THROAT | 1 |  |  | 1 |  |  | 2 |  |  |
| SKIN AND SUBCUTANEOUS TISSUE DISORDERS | ALOPECIA |  |  |  | 1 |  |  | 1 |  |  |
|  | PAIN OF SKIN | 2 |  |  | 3 | 1 |  | 5 | 1 |  |
|  | PRURITUS | 1 |  |  | 2 |  |  | 3 |  |  |
|  | RASH ACNEIFORM |  |  |  | 1 |  |  | 1 |  |  |
|  | RASH MACULO-PAPULAR | 1 |  |  | 2 | 1 |  | 3 | 1 |  |
|  | SKIN HYPERPIGMENTATION | 1 |  |  |  |  |  | 1 |  |  |
|  | SKIN HYPOPIGMENTATION | 1 |  |  |  |  |  | 1 |  |  |
|  | SKIN INDURATION | 9 | 4 |  |  | 3 |  | 9 | 7 |  |
|  | SKIN ULCERATION |  | 1 |  |  | 1 | 1 |  | 2 | 1 |
| VASCULAR DISORDERS | FLUSHING | 3 |  |  | 2 |  |  | 5 |  |  |
|  | HOT FLASHES | 3 |  |  | 2 |  |  | 5 |  |  |

**Supplemental Table 5: Direct ELIspot: number of patients in each study group with T cell response through week 26.**

| T cell response to: | Cohort |  | | | | |
| --- | --- | --- | --- | --- | --- | --- |
|  |  | Vaccine adjuvant | IFA V0 | IFA V1 | IFA V6 | IFA 0-V6 |
| N evaluable patients | 1 | LPS 25 | 3 | 2 | 2 | 7 |
|  |  | LPS 100 | 2 | 2 | 2 | 6 |
|  |  | LPS 400 | 2 | 2 | 2 | 6 |
|  |  | LPS 1600 | 4 | 4 | 6 | 14 |
|  |  | *LPS (all)* | *11* | *10* | *12* | *33* |
|  | 2 | pICLC | 6 | 6 | 6 | 18 |
|  | 1+2 | *All* | *17* | *16* | *18* | *51* |
| 12MP pool | 1 | LPS 25 | 0% | 50% | 100% | 43% |
|  |  | LPS 100 | 0% | 100% | 100% | 67% |
|  |  | LPS 400 | 50% | 50% | 50% | 50% |
|  |  | LPS 1600 | 25% | 0% | 50% | 29% |
|  |  | *LPS (all)* | *18%* | *40%* | *67%* | *42%* |
|  | 2 | pICLC | 17% | 67% | 83% | 56% |
|  | 1+2 | *All* | *18%* | *50%* | *72%* | *47%* |
| any of 12 individual peptides | 1 | LPS 25 | 0% | 50% | 100% | 43% |
|  |  | LPS 100 | 0% | 0% | 0% | 0% |
|  |  | LPS 400 | 0% | 0% | 50% | 17% |
|  |  | LPS 1600 | 0% | 0% | 33% | 14% |
|  |  | *LPS (all)* | *0%* | *10%* | *42%* | *18%* |
|  | 2 | pICLC | 17% | 67% | 83% | 56% |
|  | 1+2 | *All* | *6%* | *31%* | *56%* | *31%* |
| tetanus helper peptide | 1 | LPS 25 | 0% | 50% | 100% | 43% |
|  |  | LPS 100 | 0% | 100% | 100% | 67% |
|  |  | LPS 400 | 0% | 50% | 100% | 50% |
|  |  | LPS 1600 | 25% | 75% | 83% | 64% |
|  |  | *LPS (all)* | *9%* | *70%* | *92%* | *58%* |
|  | 2 | pICLC | 50% | 83% | 83% | 72% |
|  | 1+2 | *All* | *24%* | *75%* | *89%* | *63%* |

**Supplemental Table 6: Patient information, treatment regimen and ELIspot responses summarized per patient. Yes/no response based on positive response for one or more time points post start of vaccine.**

| **Patient information** | | | | **Vaccine adjuvants** | | | **Direct ELIspot response** | | **Stimulated ELIspot response** | |
| --- | --- | --- | --- | --- | --- | --- | --- | --- | --- | --- |
| **ID** | **Age** | **Sex** | **HLA type** | **IFA group** | **TLR agonist** | **Dose** | **12MP** | **Tetanus** | **PBMC** | **SIN** |
| 1 | 28 | M | A3 | V0 | LPS | 25 EU | no | no | yes | no |
| 2 | 62 | **F** | A1, A2 | V0 | LPS | 25 EU | no | no | yes | no |
| 3 | 61 | M | A2 | V0 | pICLC | 1 mg | no | no | yes | no |
| 5 | 45 | M | A2, A11 | V0 | LPS | 100 EU | no | no | no | ND |
| 6 | 77 | F | A1, A31 | V0 | pICLC | 1 mg | yes | yes | yes | yes |
| 7 | 78 | M | A2 | V0 | LPS | 100 EU | no | no | no | no |
| 8 | 53 | M | A2 | V1 | LPS | 25 EU | yes | yes | yes | ND |
| 9 | 79 | M | A3, A26 | V1 | LPS | 25 EU | no | no | no | no |
| 10 | 63 | M | A3 | V0 | pICLC | 1 mg | no | yes | yes | ND |
| 11 | 63 | M | A2, A3 | V0 | LPS | 400 EU | no | no | no | no |
| 12 | 65 | M | A1, A2 | V0 | LPS | 400 EU | yes | no | no | ND |
| 13 | 42 | F | A2 | V1 | pICLC | 1 mg | yes | yes | yes | yes |
| 14 | 78 | **F** | A2, A3 | V6 | LPS | 25 EU | yes | yes | yes | yes |
| 15 | 59 | M | A2 | V6 | LPS | 25 EU | yes | yes | yes | yes |
| 16 | 50 | **F** | A2 | V1 | LPS | 100 EU | yes | yes | yes | yes |
| 17 | 64 | M | A1 | V1 | LPS | 100 EU | yes | yes | yes | no |
| 18 | 61 | F | A1, A3 | V1 | pICLC | 1 mg | yes | yes | yes | yes |
| 19 | 52 | F | A1, A33 | V1 | pICLC | 1 mg | no | no | yes | yes |
| 20 | 73 | M | A2, A11 | V6 | LPS | 100 EU | yes | yes | yes | no |
| 21 | 77 | M | A31, A33 | V6 | LPS | 100 EU | yes | yes | yes | no |
| 22 | 66 | F | A1, A3 | V1 | pICLC | 1 mg | yes | yes | yes | yes |
| 23 | 52 | M | A2, A11 | V1 | pICLC | 1 mg | yes | yes | yes | no |
| 24 | 67 | **F** | A11, A23 | V1 | LPS | 400 EU | no | no | yes | no |
| 25 | 58 | M | A2, A24 | V1 | LPS | 400 EU | yes | yes | yes | no |
| 26 | 66 | F | A11, A24 | V1 | pICLC | 1 mg | no | yes | yes | no |
| 27 | 53 | M | A1 | V0 | LPS | 1600 EU | no | no | yes | yes |
| 28 | 55 | M | A1, A3 | V0 | LPS | 1600 EU | yes | no | no | no |
| 29 | 31 | M | A1 | V6 | LPS | 400 EU | yes | yes | yes | no |
| 30 | 51 | **F** | A1, A3 | V6 | LPS | 400 EU | no | yes | yes | no |
| 32 | 55 | M | A1, A68 | V0 | LPS | 25 EU | no | no | no | no |
| 33 | 55 | M | A3 | V6 | pICLC | 1 mg | yes | yes | yes | no |
| 34 | 70 | M | A1, A25 | V1 | LPS | 1600 EU | no | yes | no | ND |
| 35 | 63 | **F** | A1, A29 | V1 | LPS | 1600 EU | no | no | no | ND |
| 37 | 42 | F | A1, A2 | V6 | pICLC | 1 mg | yes | yes | yes | ND |
| 38 | 44 | F | A2 | V6 | pICLC | 1 mg | yes | yes | yes | no |
| 39 | 37 | M | A1, A2 | V0 | LPS | 1600 EU | no | yes | yes | no |
| 40 | 64 | M | A2, A23 | V6 | LPS | 1600 EU | yes | yes | yes | ND |
| 41 | 61 | M | A1, A36 | V6 | LPS | 1600 EU | yes | yes | yes | no |
| 42 | 67 | M | A2, A24 | V1 | LPS | 1600 EU | no | yes | no | no |
| 43 | 51 | F | A2 | V6 | pICLC | 1 mg | yes | yes | yes | yes |
| 44 | 61 | M | A1, A3 | V6 | LPS | 1600 EU | yes | yes | yes | ND |
| 45 | 59 | M | A11, A30 | V6 | LPS | 1600 EU | no | yes | no | no |
| 46 | 64 | M | A11, A24 | V6 | pICLC | 1 mg | yes | yes | yes | ND |
| 47 | 63 | F | A1, A24 | V6 | pICLC | 1 mg | no | no | yes | ND |
| 48 | 71 | M | A2, A33 | V6 | LPS | 1600 EU | yes? | yes | no | ND |
| 49 | 62 | M | A2, A24 | V6 | LPS | 1600 EU | no | no | yes | ND |
| 50 | 75 | F | A2 | V0 | pICLC | 1 mg | no | yes | yes | yes |
| 51 | 63 | M | A1, A3 | V0 | LPS | 1600 EU | no | no | no | ND |
| 52 | 72 | M | A2, A3 | V0 | pICLC | 1 mg | no | no | yes | ND |
| 53 | 76 | F | A1, A3 | V0 | pICLC | 1 mg | no | no | no | ND |
| 54 | 67 | **F** | A1, A2 | V1 | LPS | 1600 EU | no | yes | no | ND |
| ND = not done. Participants 4, 31, 36 not evaluable and not listed. | | | | | | | | | | |

**Supplemental Table 7. HLA expression by study group and subgroup, and associated immune response rates through week 26.**

| Distribution of HLA by study group and subgroup* | | | | | |
| --- | --- | --- | --- | --- | --- |
| N | Cohort (adjuvant) | HLA-A1 | HLA-A2 | HLA-A3 | A11,31 |
| 33 | 1a-c (LPS) | 15 (45%) | 17 (52%) | 8 (24%) | 5 (15%) |
| 18 | 2a-c (polyICLC) | 7 (39%) | 8 (44%) | 6 (33%) | 4 (22%) |
| 17 | 1a + 2a: IFA V0 | 9 (53%) | 9 (53%) | 7 (41%) | 2 (12%) |
| 16 | 1b + 2b: IFA V1 | 7 (44%) | 7 (44%) | 3 (19%) | 3 (19%) |
| 18 | 1c + 2c : IFA V6 | 6 (33%) | 9 (50%) | 4 (22%) | 4 (22%) |
| Immune response rates, ex vivo | 12MP (CD8) | 45% | 52% | 43% | 56% |
|  | HLA specific 12MP (CD8) | 14% | 36% | 29% | 33% |
|  | Any of 12MP (CD8) | 23% | 40% | 29% | 33% |
|  | Tetanus (CD4) | 55% | 68% | 50% | 78% |
| * Totals can exceed 100% because there are two HLA-A alleles each. | | | | | |

**Supplemental Table 8. Stimulated ELIspot response rate by cohort (N and %) through week 26**

| Groups |  | **DAEK** | **EVD** | **EAD** | **SSD** | **YMD** | **GLY** | **IMD** | **YLE** | **ALLA** | **SLF** | **LIY** | **ASG** |
| --- | --- | --- | --- | --- | --- | --- | --- | --- | --- | --- | --- | --- | --- |
|  | Relevant HLA | **A1** | | | | **A2** | | | | **A3, A11, A31** | | | |
| All patients  N = 51 | N (relevant HLA) | 22 | 22 | 22 | 22 | 25 | 25 | 25 | 25 | 23 | 23 | 23 | 23 |
|  | # positive | 13 | 6 | 1 | 0 | 7 | 13 | 17 | 0 | 4 | 10 | 6 | 4 |
|  | % positive | 59% | 27% | 5% | 0% | 28% | 52% | 68% | 0% | 17% | 43% | 26% | 17% |
|  |  |  |  |  |  |  |  |  |  |  |  |  |  |
| Cohort 1  (LPS)  N = 33 | N (relevant HLA) | 15 | 15 | 15 | 15 | 17 | 17 | 17 | 17 | 13 | 13 | 13 | 13 |
|  | # positive | 7 | 3 | 0 | 0 | 5 | 8 | 9 | 0 | 2 | 5 | 3 | 2 |
|  | % positive | 47% | 20% | 0% | 0% | 29% | 47% | 53% | 0% | 15% | 38% | 27% | 15% |
|  |  |  |  |  |  |  |  |  |  |  |  |  |  |
| Cohort 2  (pICLC)  N = 18 | N (relevant HLA) | 7 | 7 | 7 | 7 | 8 | 8 | 8 | 8 | 10 | 10 | 10 | 10 |
|  | # positive | 6 | 3 | 1 | 0 | 2 | 5 | 8 | 0 | 2 | 5 | 3 | 2 |
|  | % positive | 86% | 42% | 14% | 0% | 25% | 63% | 100% | 0% | 20% | 50% | 30% | 20% |

The 12MP, listed in this table, are abbreviated with the first 3-4 letters of the single-letter abbreviation codes.

**Supplemental Table 9. T cell response by IVS ELIspot to 12MP at day 183, among evaluable patients, by HLA type**

| IFA use  In vaccines | HLA-A1+ | HLA-A2+ | HLA-A3  superfamily+ | All* |
| --- | --- | --- | --- | --- |
| V0 | 2/8 (25%) | 1/7 (14%) | 1/7 (14%) | 2/14 (14%) |
| V1 | 3/6 (50%) | 3/5 (60%) | 3/5 (60%) | 6/12 (50%) |
| V6 | 4/4 (100%) | 6/7 (86%) | 5/7 (71%) | 12/14 (86%) |
| All | 9/18 (50%) | 10/19 (53%) | 9/19 (47%) | 20/40 (50%) |

* values for “All” are less than the sum across all HLA types because some patients expressed 2 different HLA alleles in these categories.

**
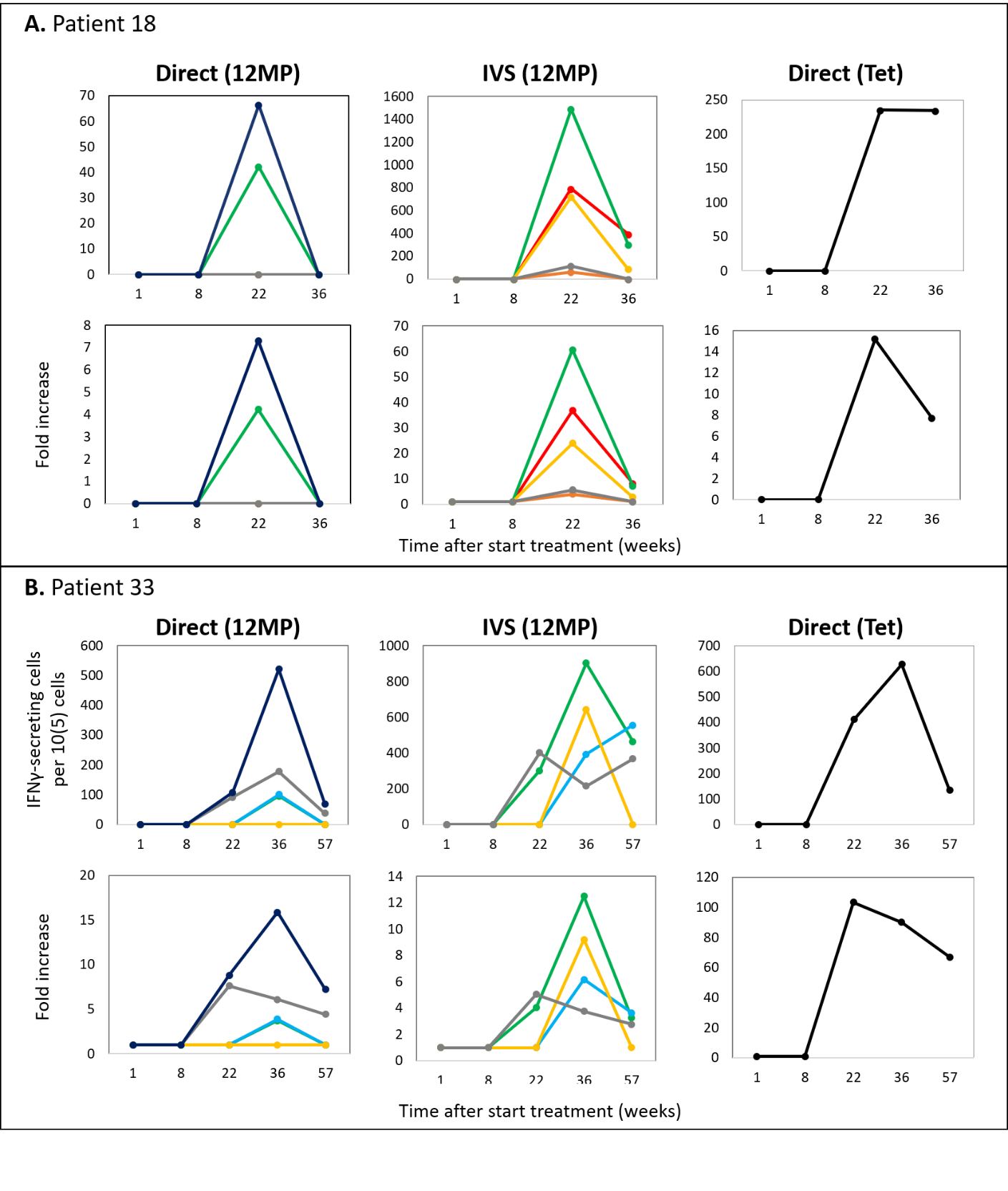
**

**Supplemental Figure 1. Immune responses in patients with DLTs.** T cell responses to 12MP peptides and to tetanus helper peptide were observed in both patients who discontinued early for DLTs, as shown for patient 18 (A,B,E,F,I,J) and 33 (C,D,G,H,K,L), with the increase response magnitudes shown as number of IFN-gamma secreting cells per 10^5^ CD8 (A-H) for the short peptides, or per 10^5^ CD4 (I-L) for the tetanus helper peptide. Data are shown both for Direct (ex vivo) ELISpot assays (A-D) for 12MP and Tetanus (I-L) and for IVS (stimulated) ELIspot assays (E-H) for 12MP. Peptide data are color coded: DAEK (red), EVD (orange), ALLA (green), LIY (yellow), SLF (gray), ASG (light blue), 12MP pool (dark blue), Tet (black).


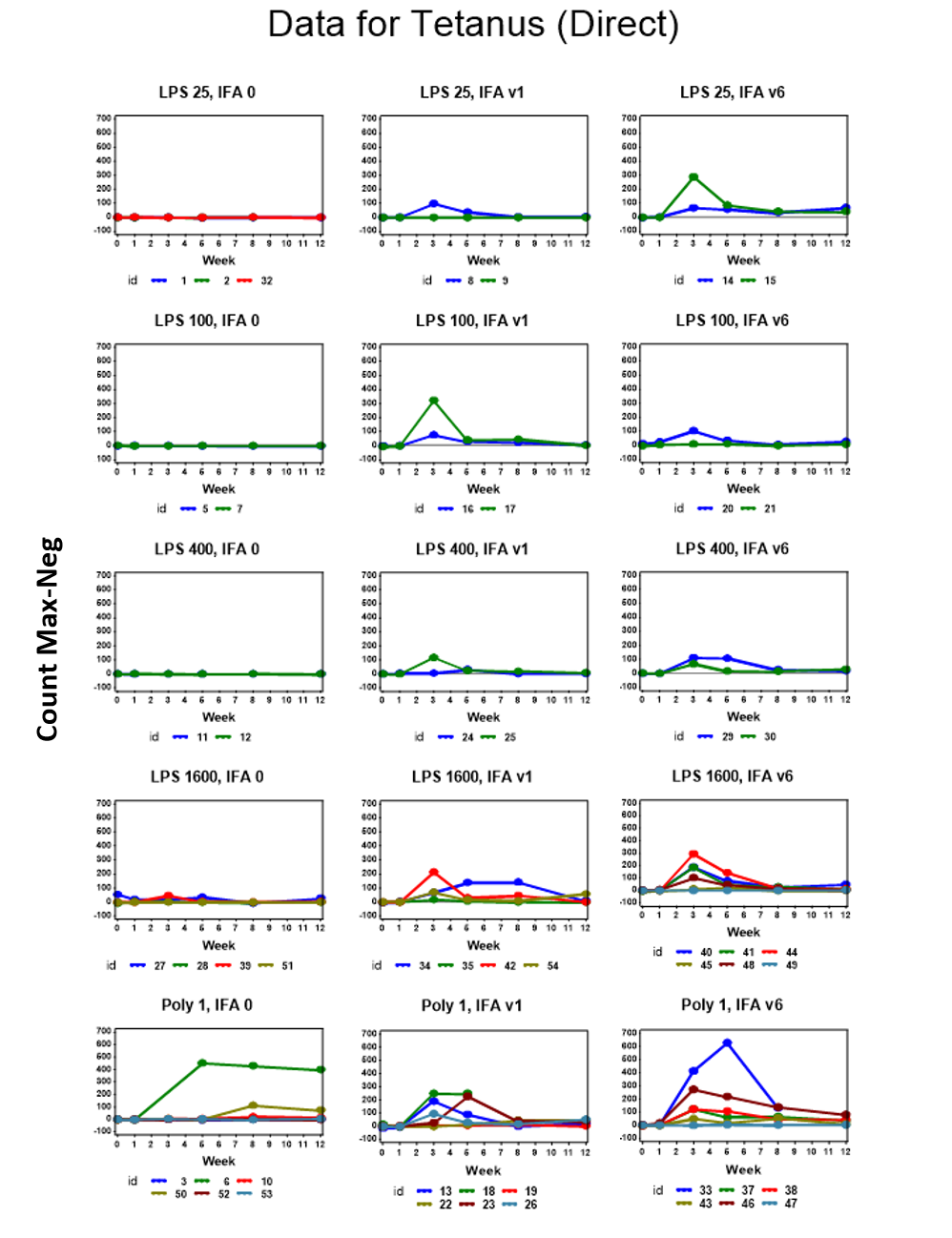


**Supplemental Figure 2.** T cell responses to tetanus peptide are shown from Direct ELIspot assay, with counts shown as IFN-gamma secreting cells per 10^~~5~~^ CD4 T cells, for each patient group, through week 12.


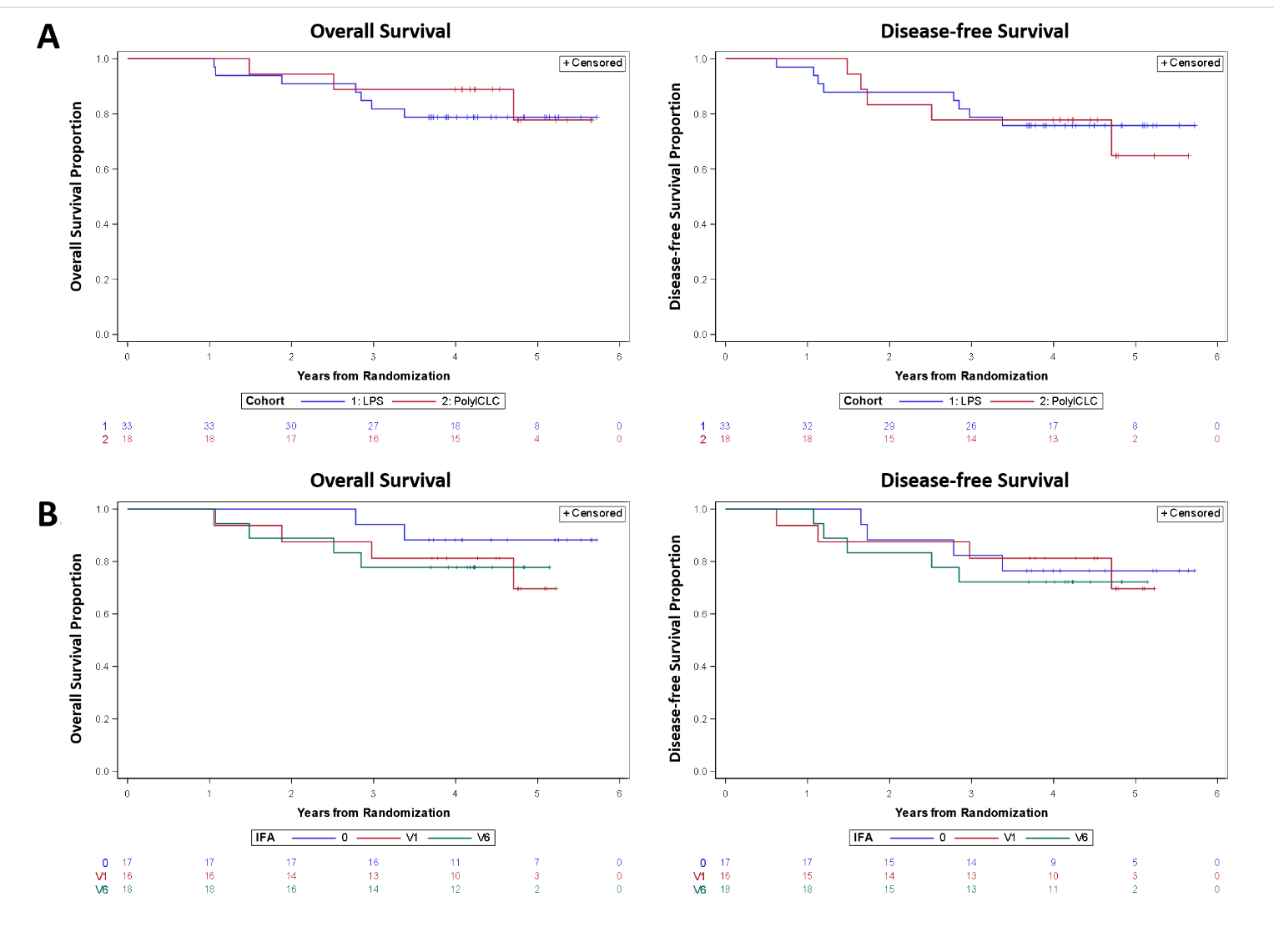


**Supplemental Figure 3.**  Survival and progression free survival curves, comparing cohort 1 and 2 (A) and IFA groups V0, V1 and V6 (B).
