# Supplementary material for: A multipeptide vaccine plus toll-like receptor agonists LPS or polyICLC in combination with incomplete Freund’s adjuvant in melanoma patients
Source: J Immunother Cancer. 2019 Jun 27;7:163. doi: 10.1186/s40425-019-0625-x (PMC6598303; doi:10.1186/s40425-019-0625-x)
Supplement: Supplementary file 2 — Supplemental Text. (DOCX 38 kb) [file 40425_2019_625_MOESM2_ESM.docx]

Supplemental Text: Methods

Study design

This was a phase I trial designed to determine the maximum tolerated dose (MTD) of the combination of LPS and IFA in cohort 1, and the maximum tolerated dose among a single dose of polyICLC and IFA in cohort 2. After determination of the MTD in each cohort, additional patients were to be accrued in order to obtain preliminary data on immune response. Patients were randomized to cohort 1 and cohort 2 in a 2:1 ratio.

Cohort 1 (LPS as TLR agonist), Phase I

*Objective:* To determine the highest dose of the combination of intradermal and subcutaneous injection of lipopolysaccharide (LPS) as a vaccine adjuvant with a multipeptide vaccine and IFA.

*Design:*  The primary goal of the dose-finding phase was to find a combination of agents with acceptable toxicity. Dose escalation was conducted using the two-stage method for dose finding with combinations of agents described in Wages, Conaway and O’Quiqley[1].

The combinations were labeled as follows:

| **Combination labels** | | | |
| --- | --- | --- | --- |
| LPS | IFA* | | |
|  | V0 | V1 | V6 |
| 25 | 1 (start) | 2 | 3 |
| 100 | 4 | 5 | 6 |
| 400 | 7 | 8 | 9 |
| 1600 | 10 | 11 | 12 |

* For this table and for the following sections, the following definitions apply:

LPS = lipopolysaccharide: the dose levels of 25, 100, 400, and 1600 EU are specified here.

IFA = incomplete Freund’s adjuvant, which in this trial is Montanide ISA-51 (Seppic, Inc.)

The subgroups related to IFA:

- 0 = subgroup 1a, where IFA is not administered with any of the 6 vaccines,
- V1 = subgroup 1b, where IFA is administered just with the first vaccine (V1), and

| **Zone** | **Drug Combinations** |
| --- | --- |
| A | 1 |
| B | 2,4 |
| C | 3,5,7 |
| D | 6,8,10 |
| E | 9,11 |
| F | 12 |

- V6 = subgroup 1c, where IFA is administered with all 6 vaccines (V6), where those subgroups are specified in Table 1 of the Protocol Precis.

For escalation decisions, subjects were observed for a minimum of three weeks after their initial dose of LPS for DLT determination.

**First stage:** The first stage treated patients in groups of size 2 until a patient experienced a dose limiting toxicity (DLT). At that point, the modeling method for stage 2 began. The escalation plan for the first stage was based on grouping dose combinations into “zones.” With this dose escalation design, patients could be accrued and assigned to other open combinations within a zone but escalation would not occur outside the zone until the minimum follow-up period was observed for the first 2 patients accrued to a combination. Note, Zone A is a single combination so additional groups of size 2 patients were not accrued until a DLT was observed or the required time frame had elapsed.

The first 2 patients were entered onto dose combination 1 (Zone A). If neither patient experienced a DLT, escalation was to proceed to Zone B, with combination 2 or 4 chosen at random. If the patients did not experience a DLT, the next group of patients were treated on the combination in Zone B that had not yet been tried. Escalation to a higher zone occurred only when all dose combinations in the lower zone had been tried, and no DLT had been observed.

**Second stage:** The second stage modeling used a selected set of possible orderings, from least to greatest, of the toxicity probabilities and a working model for the probabilities of toxicity under each ordering.

| **Orderings** | **Working models*** |
| --- | --- |
| 1-2-3-4-5-6-7-8-9-10-11-12 | 0.01-0.02-0.03-0.04-0.05-0.06-0.10-0.14-0.20-0.26-0.33-0.40 |
| 1-2-4-3-5-7-6-8-10-9-11-12 | 0.01-0.02-0.04-0.03-0.05-0.10-0.06-0.14-0.26-0.20-0.33-0.40 |
| 1-4-2-7-5-3-10-8-6-11-9-12 | 0.01-0.03-0.06-0.02-0.05-0.20-0.04-0.14-0.33-0.10-0.26-0.40 |
| 1-4-7-10-2-5-8-11-3-6-9-12 | 0.01-0.05-0.20-0.02-0.06-0.26-0.03-0.10-0.33-0.04-0.14-0.40 |
| 1-2-4-7-5-3-6-8-10-11-9-12 | 0.01-0.02-0.06-0.03-0.05-0.10-0.04-0.14-0.33-0.20-0.26-0.40 |
| 1-4-2-3-5-7-10-8-6-9-11-12 | 0.01-0.03-0.04-0.02-0.05-0.20-0.06-0.14-0.26-0.10-0.33-0.40 |
| - These working models were chosen according to the algorithm of Lee and Cheung (2009).   Because there are many dose levels, the lowest levels had working models with values of 0.  We adjusted these to have small, non-zero values. | |

Within each ordering, the continual reassessment model (CRM) was fit, using the working model and the accumulated data. The next patient was allocated to the dose indicated by the CRM within the ordering with the largest likelihood.

**Stopping rules were specified as follows:**

1. The design will stop at stage 1 at the lowest dose combination, “1”, if a single DLT is observed within the first two patients treated at that dose and declare all combinations too toxic.
2. The design will stop at the end of stage 1 if escalation proceeds to the highest dose combination, “12”, and 6 patients are treated with no DLTs at level “12”. In this case, the study is stopped and dose combination “12” is declared the MTD.
3. In the second stage, if the recommendation is to assign the next patient to a combination that already has 6 patients treated on the combination, the study is stopped and the recommended combination is declared the MTD.
4. Occurrence of grade 4 or greater treatment-related toxicity will prompt the investigator to conduct a thorough evaluation of the available safety information to decide whether to continue accruing new subjects into the study.
5. The study of cohort 1 will proceed to a maximum of 54 patients. The MTD is the combination that would be recommended for the 55^th^ patient.

**Statistical properties:** Simulations were run to display the performance of the design characteristics for cohort sizes of 2. For each of the scenarios, 1000 simulated trials were run.

| \| **Scenario 1** \| \| \| \| \| \| --- \| --- \| --- \| --- \| --- \| \|  \|  \| % Recommended  as MTD with  cohort size = 2 \| \| \| \| Truth \| LPS \|  \| IFA \|  \| \|  \| 0 \| V1 \| V6 \| \|  \| 25 \| 1% \| 2% \| 4% \| \|  \| 100 \| 2% \| 4% \| 6% \| \|  \| 400 \| 4% \| 6% \| 8% \| \|  \| 1600 \| 8% \| 10% \| **12%** \| \| Simulations (1000) \|  \|  \|  \|  \| \| % recommended as the MTD \| 25 \| 0% \| 0% \| 2% \| \|  \| 100 \| 0% \| 0% \| 4% \| \|  \| 400 \| 0% \| 4% \| 8% \| \|  \| 1600 \| 5% \| 12% \| *62%* \| \|  \|  \|  \|  \|  \| \| % patients allocated to a given dose \| 25 \| 7% \| 7% \| 7% \| \|  \| 100 \| 7% \| 6% \| 7% \| \|  \| 400 \| 7% \| 7% \| 7% \| \|  \| 1600 \| 7% \| 16% \| 14% \| \|  \|  \|  \|  \|  \| \| % recommended dose ± 5% target rate \|  \|  \|  \| 62% \| \| % stopped after 1^st^ cohort \|  \|  \|  \| 1.8% \| \| Average trial size \|  \|  \|  \| 28.6 \| \| Average % of DLTs \|  \|  \|  \| 6.2% \| \| Target rate for MTD 20%  Appropriate dose(s) **(**within ± 5% target rate) denoted in **bold**  Recommended dose(s) (within ± 5% target rate) denoted in *italics* \| \| \| \| \| | \| **Scenario 2** \| \| \| \| \| \| --- \| --- \| --- \| --- \| --- \| \|  \|  \| % Recommended  as MTD with  cohort size = 2 \| \| \| \| Truth \| LPS \|  \| IFA \|  \| \|  \| 0 \| V1 \| V6 \| \|  \| 25 \| 3% \| 6% \| 12% \| \|  \| 100 \| 8% \| 14% \| **20%** \| \|  \| 400 \| **16%** \| **22%** \| 28% \| \|  \| 1600 \| **24%** \| 30% \| 36% \| \| Simulations (1000) \|  \|  \|  \|  \| \| % recommended as the MTD \| 25 \| 1% \| 2% \| 7% \| \|  \| 100 \| 2% \| 6% \| *15%* \| \|  \| 400 \| *11%* \| *20%* \| 8% \| \|  \| 1600 \| *10%* \| 7% \| 3% \| \|  \|  \|  \|  \|  \| \| % patients allocated to a given dose \| 25 \| 9% \| 10% \| 9% \| \|  \| 100 \| 10% \| 9% \| 10% \| \|  \| 400 \| 11% \| 11% \| 6% \| \|  \| 1600 \| 8% \| 6% \| 2% \| \|  \|  \|  \|  \|  \| \| % recommended dose ± 5% target rate \|  \|  \|  \| 56% \| \| % stopped after 1^st^ cohort \|  \|  \|  \| 6.5% \| \| Average trial size \|  \|  \|  \| 22.8 \| \| Average % of DLTs \|  \|  \|  \| 15.6% \| \| Target rate for MTD 20%  Appropriate dose(s) **(**within ± 5% target rate) denoted in **bold**  Recommended dose(s) (within ± 5% target rate) denoted in *italics* \| \| \| \| \| |
| --- | --- | --- | --- | --- | --- | --- | --- | --- | --- | --- | --- | --- | --- | --- | --- | --- | --- | --- | --- | --- | --- | --- | --- | --- | --- | --- | --- | --- | --- | --- | --- | --- | --- | --- | --- | --- | --- | --- | --- | --- | --- | --- | --- | --- | --- | --- | --- | --- | --- | --- | --- | --- | --- | --- | --- | --- | --- | --- | --- | --- | --- | --- | --- | --- | --- | --- | --- | --- | --- | --- | --- | --- | --- | --- | --- | --- | --- | --- | --- | --- | --- | --- | --- | --- | --- | --- | --- | --- | --- | --- | --- | --- | --- | --- | --- | --- | --- | --- | --- | --- | --- | --- | --- | --- | --- | --- | --- | --- | --- | --- | --- | --- | --- | --- | --- | --- | --- | --- | --- | --- | --- | --- | --- | --- | --- | --- | --- | --- | --- | --- | --- | --- | --- | --- | --- | --- | --- | --- | --- | --- | --- | --- | --- | --- | --- | --- | --- | --- | --- | --- | --- | --- | --- | --- | --- | --- | --- | --- | --- | --- | --- | --- | --- | --- | --- | --- | --- | --- | --- | --- | --- | --- | --- | --- | --- | --- | --- | --- | --- | --- | --- | --- | --- | --- | --- | --- | --- | --- | --- | --- | --- | --- | --- | --- | --- | --- | --- | --- | --- | --- | --- | --- | --- | --- | --- | --- | --- | --- | --- | --- | --- | --- | --- | --- | --- | --- | --- | --- | --- | --- | --- | --- | --- | --- | --- | --- | --- | --- | --- | --- | --- | --- | --- | --- | --- | --- | --- | --- | --- |
| \| **Scenario 3** \| \| \| \| \| \| --- \| --- \| --- \| --- \| --- \| \|  \|  \| % Recommended  as MTD with  cohort size = 2 \| \| \| \| Truth \| LPS \|  \| IFA \|  \| \|  \| 0 \| V1 \| V6 \| \|  \| 25 \| **15%** \| **20%** \| **25%** \| \|  \| 100 \| **20%** \| **25%** \| 35% \| \|  \| 400 \| 30% \| 35% \| 60% \| \|  \| 1600 \| 45% \| 55% \| 75% \| \| Simulations (1000) \|  \|  \|  \|  \| \| % recommended as the MTD \| 25 \| *24%* \| *9%* \| *6%* \| \|  \| 100 \| *10%* \| *5%* \| 5% \| \|  \| 400 \| 8% \| 4% \| 0% \| \|  \| 1600 \| 2% \| 0% \| 0% \| \|  \|  \|  \|  \|  \| \| % patients allocated to a given dose \| 25 \| 21% \| 14% \| 9% \| \|  \| 100 \| 15% \| 10% \| 7% \| \|  \| 400 \| 11% \| 6% \| 1% \| \|  \| 1600 \| 4% \| 2% \| 0% \| \|  \|  \|  \|  \|  \| \| % recommended dose ± 5% target rate \|  \|  \|  \| 54% \| \| % stopped after 1^st^ cohort \|  \|  \|  \| 26.7% \| \| Average trial size \|  \|  \|  \| 15.1 \| \| Average % of DLTs \|  \|  \|  \| 24.9% \| \| Target rate for MTD 20%  Appropriate dose(s) **(**within ± 5% target rate) denoted in **bold**  Recommended dose(s) (within ± 5% target rate) denoted in *italics* \| \| \| \| \| | \| **Scenario 4** \| \| \| \| \| \| --- \| --- \| --- \| --- \| --- \| \|  \|  \| % Recommended  as MTD with  cohort size = 2 \| \| \| \| Truth \| LPS \|  \| IFA \|  \| \|  \| 0 \| V1 \| V6 \| \|  \| 25 \| **25%** \| 40% \| 50% \| \|  \| 100 \| 40% \| 55% \| 60% \| \|  \| 400 \| 50% \| 65% \| 70% \| \|  \| 1600 \| 60% \| 70% \| 80% \| \| Simulations (1000) \|  \|  \|  \|  \| \| % recommended as the MTD \| 25 \| *46%* \| 2% \| 0% \| \|  \| 100 \| 3% \| 0% \| 0% \| \|  \| 400 \| 0% \| 0% \| 0% \| \|  \| 1600 \| 0% \| 0% \| 0% \| \|  \|  \|  \|  \|  \| \| % patients allocated to a given dose \| 25 \| 49% \| 17% \| 4% \| \|  \| 100 \| 17% \| 4% \| 2% \| \|  \| 400 \| 6% \| 1% \| 0% \| \|  \| 1600 \| 2% \| 0% \| 0% \| \|  \|  \|  \|  \|  \| \| % recommended dose ± 5% target rate \|  \|  \|  \| 49% \| \| % stopped after 1^st^ cohort \|  \|  \|  \| 48.1% \| \| Average trial size \|  \|  \|  \| 7.9 \| \| Average % of DLTs \|  \|  \|  \| 35.2% \| \| Target rate for MTD 20%  Appropriate dose(s) **(**within ± 5% target rate) denoted in **bold**  Recommended dose(s) (within ± 5% target rate) denoted in *italics* \| \| \| \| \| |

Cohort 2 (PolyICLC as TLR agonist), Phase I

*Objective:* To determine the highest dose of IFA that can be given with Poly ICLC at a fixed dose level.

*Design:*  The primary goal of the dose-finding phase is to find the MTD among the 3 possible dose levels. With only 3 possible dose levels the traditional 3+3 design was employed.

| Poly ICLC | IFA* | | |
| --- | --- | --- | --- |
|  | V0 | V1 | V6 |
| 1 mg | Level 1 | Level 2 | Level 3 |

To determine the MTD: We treated patients in groups of size 3 until a patient experienced a dose limiting toxicity (DLT). If 0/3 patients experienced a DLT, then the next group was treated at the next higher level. If 1/3 patients experienced a DLT then 3 additional patients are treated at the current level. Escalation was to continue if 1/6 patients experience a DLT. If ≥2/3 patients experienced a DLT, then additional patients would be treated at the lower level if fewer than 6 patients had been treated at that level. The MTD is the highest level where ≤1/6 patients experience a DLT.

Expansion: In order to assess the impact of including IFA (or not) on the immunologic parameters it is of interest to accrue additional patients at the highest levels of LPS and Poly ICLC considered safe for each level of IFA (i.e., the highest level where 0, V1 or V6 was administered safely). Therefore, we will randomly back fill those dose levels until 6 patients have been treated at each dose combination of interest. This results in 3 combinations for cohort 1 and 3 for cohort 2 if the MTD is the highest level. The choice of 6 patients per final combination was chosen to mimic the standard phase I decision rule and to provide better estimates of variability for the immunologic parameters of interest.

Overall accrual for both cohorts: Accrual was estimated at 4 patients per month. In the unanticipated event that both phase I portions require 6 patients per dose combination then the maximum possible target accrual for this study would be 72 patients. However, it is anticipated that very few, if any, DLTs will be observed within either cohort. Therefore, it is assumed that cohort 1 will require approximately 22 (average trial size from simulations 1-3) + 8 (to back fill). We anticipate also that cohort 2 will accrue 18 (including back fill), for an estimated target accrual of 48 patients. Adjusting for a 5% ineligibility/dropout rate then target accrual is estimated at 51 patients.

References Cited:

1. Wages NA, Conaway MR, O'Quigley J. Dose-finding design for multi-drug combinations. Clin. Trials 2011; 8: 380-389.
